# Nitric oxide sensor NsrR is the key direct regulator of magnetosome formation and nitrogen metabolism in *Magnetospirillum*

**DOI:** 10.1093/nar/gkad1230

**Published:** 2024-01-09

**Authors:** Bo Pang, Haolan Zheng, Shijia Ma, Jiesheng Tian, Ying Wen

**Affiliations:** State Key Laboratory of Animal Biotech Breeding and College of Biological Sciences, China Agricultural University, Beijing 100193, China; State Key Laboratory of Animal Biotech Breeding and College of Biological Sciences, China Agricultural University, Beijing 100193, China; State Key Laboratory of Animal Biotech Breeding and College of Biological Sciences, China Agricultural University, Beijing 100193, China; State Key Laboratory of Animal Biotech Breeding and College of Biological Sciences, China Agricultural University, Beijing 100193, China; State Key Laboratory of Animal Biotech Breeding and College of Biological Sciences, China Agricultural University, Beijing 100193, China

## Abstract

Nitric oxide (NO) plays an essential role as signaling molecule in regulation of eukaryotic biomineralization, but its role in prokaryotic biomineralization is unknown. *Magnetospirillum gryphiswaldense* MSR-1, a model strain for studies of prokaryotic biomineralization, has the unique ability to form magnetosomes (magnetic organelles). We demonstrate here that magnetosome biomineralization in MSR-1 requires the presence of NsrR*_Mg_* (an NO sensor) and a certain level of NO. MSR-1 synthesizes endogenous NO via nitrification-denitrification pathway to activate magnetosome formation. NsrR*_Mg_* was identified as a global transcriptional regulator that acts as a direct activator of magnetosome gene cluster (MGC) and nitrification genes but as a repressor of denitrification genes. Specific levels of NO modulate DNA-binding ability of NsrR*_Mg_* to various target promoters, leading to enhancing expression of MGC genes, derepressing denitrification genes, and repressing nitrification genes. These regulatory functions help maintain appropriate endogenous NO level. This study identifies for the first time the key transcriptional regulator of major MGC genes, clarifies the molecular mechanisms underlying NsrR-mediated NO signal transduction in magnetosome formation, and provides a basis for a proposed model of the role of NO in the evolutionary origin of prokaryotic biomineralization processes.

## Graphical Abstract

**Graphical Abstract:**
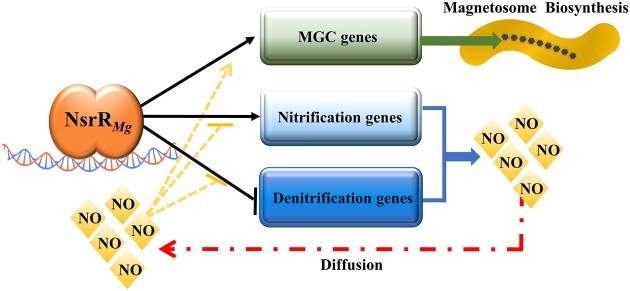

## Introduction

Nitric oxide (NO) is an important signaling molecule, widely employed among prokaryotes and eukaryotes, and plays key roles in regulation of a variety of biological processes, including NO detoxification, NO damage repair, biofilm formation, and pathogen virulence in bacteria (1–3), and vasodilation, neurotransmission, and oncogenesis in higher animals (4–6). Previous studies have demonstrated its involvement in biomineralization (mainly calcification) in eukaryotes (7,8), and in control of bone formation in mammals (9). However, the role of NO signaling in biomineralization in prokaryotes has not been investigated.

Magnetotactic bacteria (MTB) are a group of phylogenetically and morphologically diverse aquatic prokaryotes (10). They are useful model organisms for studies of biomineralization in prokaryotes, which arose in the oceans roughly 3 billion years (Gyr) ago (11), long before the appearance of eukaryotes (12). MTB have the unique ability to synthesize organelles, termed magnetosomes, composed of membrane-enveloped, nanosized, single-domain crystals of magnetite (Fe_3_O_4_) and/or greigite (Fe_3_S_4_) (13,14). MTB utilize magnetosomes for orientation along geomagnetic fields, to find redox zones favorable for growth. Direct association with magnetosome formation has been documented for >30 genes to date (15,16). Such genes are localized in a large cluster, termed ‘magnetosome gene cluster (MGC)’ or ‘magnetosome island (MAI)’, in the MTB genome. Five polycistronic operons (*mamAB*, *mamXY*, *mamGFDC*, *mms6*, *feoAB1*) have been identified in MAI of *Magnetospirillum* (class α-proteobacteria) species (17,18). *mamAB*, the core operon for basic biomineralization, consists of 17 structural genes that encode proteins essential for magnetosome formation (19,20). *mamABEIKMPQ* genes in this operon are conserved in studied/known MTB species (21,22). The other four operons above encode auxiliary proteins for magnetosome biomineralization; *mamXY*, *mamGFDC* and *mms6* regulate size and shape of core magnetite crystals, and *feoAB1* is involved in transportation of iron ions (23–25). Our knowledge regarding detailed molecular mechanisms of biomineralization and its regulation remains fragmentary.

Magnetosome biomineralization is under complex, precise control in response to environmental and physiological changes. Magnetite biomineralization and crystallization depend on overall redox balance within the magnetosome vesicle and host cell, whereby co-precipitation occurs for a certain ratio of ferric (Fe^3+^) and ferrous (Fe^2+^) ions. Iron-responsive regulators Fur and IrrB regulate genes involved in iron metabolism, and determine magnetosome size and number in the well-studied model MTB strain *Magnetospirillum gryphiswaldense* MSR-1 (26,27). Magnetosome biomineralization in all of known MTB is inhibited by high oxygen (O_2_) partial pressure in the environment. The nature of MTB magnetotaxis is considered as flagellum-based aerotaxis toward microaerobic or anaerobic environments with the aid of geomagnetic field (28). Attempts to identify magnetosome biosynthesis regulators were accordingly focused on regulators responsive to O_2_ or peroxides (molecules in which two oxygen atoms are linked by a single covalent bond). However, O_2_-responsive MgFnr (29), peroxide-responsive OxyR (30), and its homolog OxyR-like (31) were all shown not to directly regulate *mamAB* operon, although OxyR regulates expression of *mamGFDC*, *mamXY* and *feoAB1* operons (30). To date, no direct transcriptional regulator of the most important *mamAB* operon has been identified.

Genes outside MAI also affect magnetosome formation (25,26,32–34). Dissimilatory denitrification, an essential pathway for anaerobic growth of MTB (35,36), evidently helped regulate magnetosome formation in MSR-1 by maintaining intracellular redox balance. Deletion of periplasmic nitrate reductase gene (*nap*) and/or nitrite reductase gene (*nir*) in this pathway resulted in biomineralization defects (33). Regulators MgFnr and Mg2046 (DnrA) in MSR-1 were shown to affect magnetosome biomineralization indirectly through their impact on denitrification-driven redox reactions (26,34). We suggested that certain intermediate of denitrification may play some role in magnetosome formation. Among intermediates of denitrification pathway, NO is the most widely studied signaling molecule (1,3). However, its role in magnetosome biomineralization has not been reported.

At high concentrations, NO has cytotoxic effects because it forms highly reactive nitrogen intermediates (this is termed ‘nitrosative stress’). On the other hand, NO at low concentrations plays useful roles in a variety of physiological processes in both eukaryotes and prokaryotes (37,38). Numerous regulatory mechanisms have evolved for responding to NO (39). The transcriptional regulator protein NsrR is widely present as a specific NO sensor in most γ- and β-proteobacteria, which are Gram-negative (40), is also present in certain Gram-positive genera such as *Streptomyces* (41) and *Bacillus* (42), but it has not been reported in α-proteobacteria. NsrR is a member of the Rrf2 family of prokaryotic transcriptional regulators (43), characterized by presence of either a [2Fe–2S] or [4Fe–4S] cluster, depending on species and purification conditions (44,45). The major functions of NsrR are detection of NO, and regulation of expression of genes involved in NO detoxification and reparation of damage by reactive nitrogen intermediates. NsrR usually acts as a homodimeric transcriptional repressor by binding to a conserved inverted repeat sequence (43). In response to S-nitrosylation, whereby NO reacts with cysteine thiol residues on NsrR, the protein is disassociated from DNA and derepresses expression of target genes (1). Remarkably, in *Salmonella typhimurium*, NsrR functions as an activator of virulence gene expression (2,46). We found an NsrR homolog in MSR-1 and named it NsrR*_Mg_*. The function of NsrR*_Mg_* is yet unknown.

In the present study, we investigated the role of NsrR*_Mg_* in MSR-1 and demonstrated that it responds to NO signaling and directly activates expression of MGC genes – including the core *mamAB* operon. NsrR*_Mg_* is the first identified transcriptional regulator that directly regulates this operon and is required for magnetosome formation. We also demonstrated existence of a nitrification-denitrification metabolic pathway that supports endogenous NO production in MSR-1, characterized NsrR*_Mg_* as a dual repressor/activator in this pathway. These findings help to elucidate NsrR function and regulatory mechanisms in magnetosome biomineralization. Because MTB are among the oldest and simplest organisms capable of biomineralization (47), results presented here will help clarify the evolutionary origin of this process, and the intrinsic nature of magnetotaxis.

## Materials and methods

### Bacterial strains, plasmids and growth conditions

Strains and plasmids used in this study are listed in Supplementary Table S1, and primers used are listed in Supplementary Table S2. *M. gryphiswaldense* MSR-1 (DSM No. 6361) was the wild-type (WT) strain used for magnetosome synthesis. *Escherichia coli* strains DH5α, S17-1 and BL21 (DE3) were used respectively for DNA cloning, conjugation transfer, and overexpression of target proteins. MSR-1 and its derivatives were cultured in modified sodium lactate medium (SLM) (31) (termed mSLM) at 30°C with rotation (100 rpm), and microaerobic conditions developed in the medium with gradually increased cell densities due to O_2_ consumption. The medium (per liter) contained 2.25 g sodium lactate, 0.05 g sodium thioglycolate, 0.4 g NH_4_Cl, 0.5 g K_2_HPO_4_, 0.1 g MgSO_4_⋅7H_2_O, and 5 ml of trace element mixture (25). Ferric citrate (iron source) was added to mSLM at final concentration 60 μM. *E. coli* were cultured in Luria broth (LB) at 37°C. Antibiotics used for MSR-1 and *E. coli* culture were described previously (30,34).

### Construction of *nsrR_Mg_* deletion and complemented strains

*nsrR_Mg_* gene was deleted through homologous recombination. *nsrR_Mg_* deletion mutant was constructed by amplification of an 855-bp 5′-flanking region and a 921-bp 3′-flanking region from MSR-1 WT genome by PCR with respective primer pairs PB1A/PB1B and PB2A/PB2B (Supplementary Figure S1). The two fragments were digested respectively with EcoRI/BamHI and BamHI/SacI, and gentamicin (Gm)-resistant cassette was cut with BamHI from plasmid pUC-Gm (48). These three fragments were ligated simultaneously into EcoRI/SacI-digested pUX19 (49) to generate *nsrR_Mg_* deletion vector pUX-ΔnsrR*_Mg_*, which was then introduced into MSR-1 WT by conjugation with *E. coli* S17-1 as donor strain. Gm- and nalidixic acid (Nx)-resistant strains were selected, and the obtained *nsrR_Mg_* deletion mutant (termed ΔnsrR*_Mg_*) was confirmed by PCR using primer pairs PB3A/PB3B (located within deletion region), GmA/GmB (at both ends of Gm-resistant cassette), and PB4A/PB4B (flanking exchange regions) (Supplementary Figure S1).

For complementation of ΔnsrR*_Mg_*, a 321-bp *nsrR_Mg_* promoter region and a 462-bp *nsrR_Mg_* coding region were respectively amplified with primer pairs PB5A/PB5B and PB6A/PB6B. The obtained PCR products were digested respectively with NsiI/BamHI and BamHI/XbaI, and then ligated simultaneously into NsiI/XbaI-digested pBBR1MCS-2 (50) to generate *nsrR_Mg_*- complemented plasmid pMCS-CnsrR*_Mg_*, which was introduced into ΔnsrR*_Mg_* by conjugation to obtain complemented strain CnsrR*_Mg_*.

### Analysis of cell growth and magnetosome formation

Cell growth was determined based on OD_565_ of MSR-1 cultures. Magnetic response was estimated as Cmag value (coefficient of magnetically induced differential light scattering) from measurement of maximal and minimal scattering intensity, as described previously (25,51). OD_565_ and Cmag values were analyzed at 2-h intervals for construction of growth and Cmag curves.

Magnetosome formation was observed by transmission electron microscopy (TEM) (model JEM-1230, JEOL, Japan) in samples prepared as described previously (30). Statistical analysis of magnetosome numbers and diameters was performed using ImageJ software program (imagej.nih.gov/ij).

### Iron absorption capacity, intracellular and cytosolic iron content

Supernatants were taken at 6-h intervals from MSR-1 strains cultured in mSLM with 60 μM ferric citrate, and total iron ions were measured by ferrozine method for residual iron content in medium (30,52).

Cells were collected by centrifugation (12 000 rpm, 5 min) after 18 h growth, washed 3x with buffer containing 0.5 mM EDTA (pH 7.4) and 20 mM Tris–HCl, dried at 60°C to constant weight, and digested with nitric acid for 3 h at 95°C. Total intracellular iron content was measured by atomic absorption spectrometry (Optima 5300 DV system, PerkinElmer, USA).

The cytosolic iron content of MSR-1 strains was measured by an iron colorimetric assay kit (BioVision, USA). Cells were collected, and washed 3× with iron assay buffer. Following sonication and centrifugation, ferric iron in the supernatant was reduced to ferrous iron by iron reducer in the kit, and then the sample was incubated with Iron Probe at 25°C in the dark for 1 h and detected by a microplate reader (SpectraMax Plus, BioTek, USA) at 593 nm. Total cytosolic iron content was calculated based on the absorbance values of a standard concentration curve (constructed using iron standard in the kit).

### Quantitative real-time reverse transcription-PCR (qRT-PCR)

WT and ΔnsrR*_Mg_* were cultured in mSLM containing 60 μM ferric citrate for 6, 12, 18 or 24 h, and triturated samples were collected at each time point. Total RNAs were extracted with TRIzol reagent (Tiangen, China), and digested by RNase-free DNase I (TaKaRa; Japan) to remove DNA contamination. RNAs used for analysis of NO effect on gene expression were prepared from cells treated with 50 μM sodium nitroprusside (SNP; NO donor) for 18 h. Reverse transcription of total RNA (5 μg) for cDNA synthesis was performed using M-MLV reverse transcriptase (Promega, USA). Transcript levels of tested genes were quantified by qPCR using primers listed in Supplementary Table S1 and 480 SYBR Green I Master Kit (Roche, USA), and calculated by 2^−ΔΔCt^ method (53). Housekeeping gene *rpoC* (*MGMSRv2_0030*) was used as internal control and as reference for sample normalization. Experiments were performed in triplicate.

### Heterologous expression and purification of His_6_-NsrR*_Mg_*

NsrR*_Mg_* protein expression plasmid pET-28a-NsrR*_Mg_* was constructed for overexpression of N-terminal His_6_-tagged NsrR*_Mg_* in *E. coli*. The 488-bp NsrR*_Mg_* coding region was amplified from MSR-1 WT genome using primer pair PB7A/PB7B. The obtained PCR product was digested with BamHI/HindIII and cloned into corresponding sites of pET28a (+) to generate pET-28a-NsrR*_Mg_*, which was transformed into *E. coli* BL21 (DE3) for His_6_-NsrR*_Mg_* overexpression.

The transformant was cultured in 800 mL LB containing 20 μM ammonium ferric citrate and 50 μg/ml kanamycin until OD_600_ reached 0.4–0.6. Synthesis of intracellular Fe–S cluster was promoted by placing culture on ice for 18 min and adding 0.4 μM IPTG for induction. Culture was incubated at 16°C for 4 h, added with 200 μM ferric ammonium citrate and 25 μM L-methionine, and incubated for another 14 h. Cells were harvested, disrupted in 25 ml lysis buffer (300 mM NaCl, 50 mM NaH_2_PO_4_) by sonication on ice under anaerobic conditions, centrifuged, subjected to repeated vacuuming and nitrogen filling, and supernatant was transferred to an anaerobic glovebox cabinet (MBRAUN Lab Star; Germany). Soluble His_6_-NsrR*_Mg_* was purified on Ni-NTA column (Novagen; Germany) and eluted with lysis buffer plus 250 mM imidazole in the cabinet (O_2_< 20 ppm). All solutions used for anaerobic purification were filled with nitrogen for 30 min to remove O_2_, subjected to repeated vacuuming and nitrogen filling, and transferred to the cabinet.

### Electrophoretic mobility shift assays (EMSAs)

EMSAs were performed as described previously (30). 3′-digoxigenin (DIG)-labeled promoter probes were generated by PCR using respective primers listed in Supplementary Table S2. Each binding reaction mixture (20 μl) contained 1 μg poly[d(I-C)], 0.3 nM DIG-labeled probe, various amounts of His_6_-NsrR*_Mg_* in binding buffer [20 mM HEPES, 1 mM EDTA, 30 mM KCl, 10 mM (NH_4_)_2_SO_4_, 1 mM DTT], and was incubated at room temperature for 30 min. Specificity of NsrR*_Mg_*/probe interaction was confirmed by adding ∼400-fold excess of unlabeled nonspecific probe *rpoCp* or corresponding specific probe to the reaction system.

Effects of NO and O_2_ on NsrR*_Mg_* probe interaction were evaluated by adding SNP (NO donor) or sodium percarbonate (SPC; O_2_ donor) to the EMSA reaction system at various final concentrations.

### Bioluminescence assay in *E*. *coli*

Promoter regions of *mamH* (285-bp), *mamI* (202-bp) and *norC* (194-bp) were amplified with primer pairs PB21A/PB21B, PB24A/PB24B and PB31A/PB31B, and ligated into reporter plasmid pCS26-*Pac* (54) to obtain pOmamHp-lux, pOmamIp-lux and pOnorCp-lux, in which *lux* gene was controlled by *mamHp*, *mamIp*, and *norCp*, respectively. For expression of NsrR*_Mg_*, the 595-bp *nsrR_Mg_* fragment containing its open reading frame (ORF) and ribosome-binding site was amplified with primer pair PB27A/PB27B, and cloned into pACYC184 (54) to give pNsrR*_Mg_*. Control plasmid pACYC184 and NsrR*_Mg_* expression plasmid pNsrR*_Mg_* were transformed into *E. coli* DH5α bearing pOmamHp-lux, pOmamIp-lux, or pOnorCp-lux, respectively. Bioluminescence levels of reporter cultures were analyzed as described previously (55).

### Intracellular NO level

Intracellular NO level was determined using NO detection kit (Beyotime) containing diaminofluorescein-FM diacetate (DAF-FM DA). Fluorescence intensity was measured by spectrofluorometry (F-4500, Hitachi; Japan), with excitation wavelength 488 nm and emission wavelength 525 nm. MSR-1 strains were cultured in mSLM with 60 μM ferric citrate for various durations, and OD_565_ values were measured. Harvested cells were incubated with 10 μM DAF-FM DA for 1 h at 37°C in the dark, and washed 3× with PBS (pH 7.4) buffer. All samples were suspended in 1 ml PBS buffer, and NO levels were calculated as ratio of fluorescence intensity to OD_565_.

### Statistical analysis

Statistical analysis was performed using unpaired two-tailed Student's *t*-test in software program GraphPad Prism 9. Error bars shown in figures represent mean ± SD from three biological replicates.

## Results

### NsrR*_Mg_* is required for magnetosome synthesis

The gene *MGMSRv2_0820* in *M*. *gryphiswaldense* MSR-1 contains 438 nucleotides (nt) and encodes a 145-amino acid protein (termed NsrR*_Mg_*) homologous to bacterial NsrR proteins. Protein alignment revealed respective sequence identities 69%, 44% and 40% of NsrR*_Mg_* with its homologs in *Magnetospirillum magneticum* AMB-1, *Bacillus subtilis* and *E. coli*. Phylogenetic analysis showed that NsrR*_Mg_* belongs to the subfamily of NsrR-like regulators and does not cluster with the other subfamilies of Rrf2 family regulators (Supplementary Figure S2A). Although NsrR homologs are widely distributed among bacteria, they are not present in all non-magnetic bacteria, such as Pasteurellaceae, Pseudomonadales and *Vibrio cholerae* (56). BLAST search revealed presence of NsrR homologs in all genome-annotated MTB, examined from the two Pseudomonadota classes (α-, γ-proteobacteria), different genera of Thermodesulfobacteriota, Nitrospirota, Omnitrophota, and Planctomycetota (Supplementary Figure S2B), implying the importance of NsrR function in MTB.

**Figure 1.**
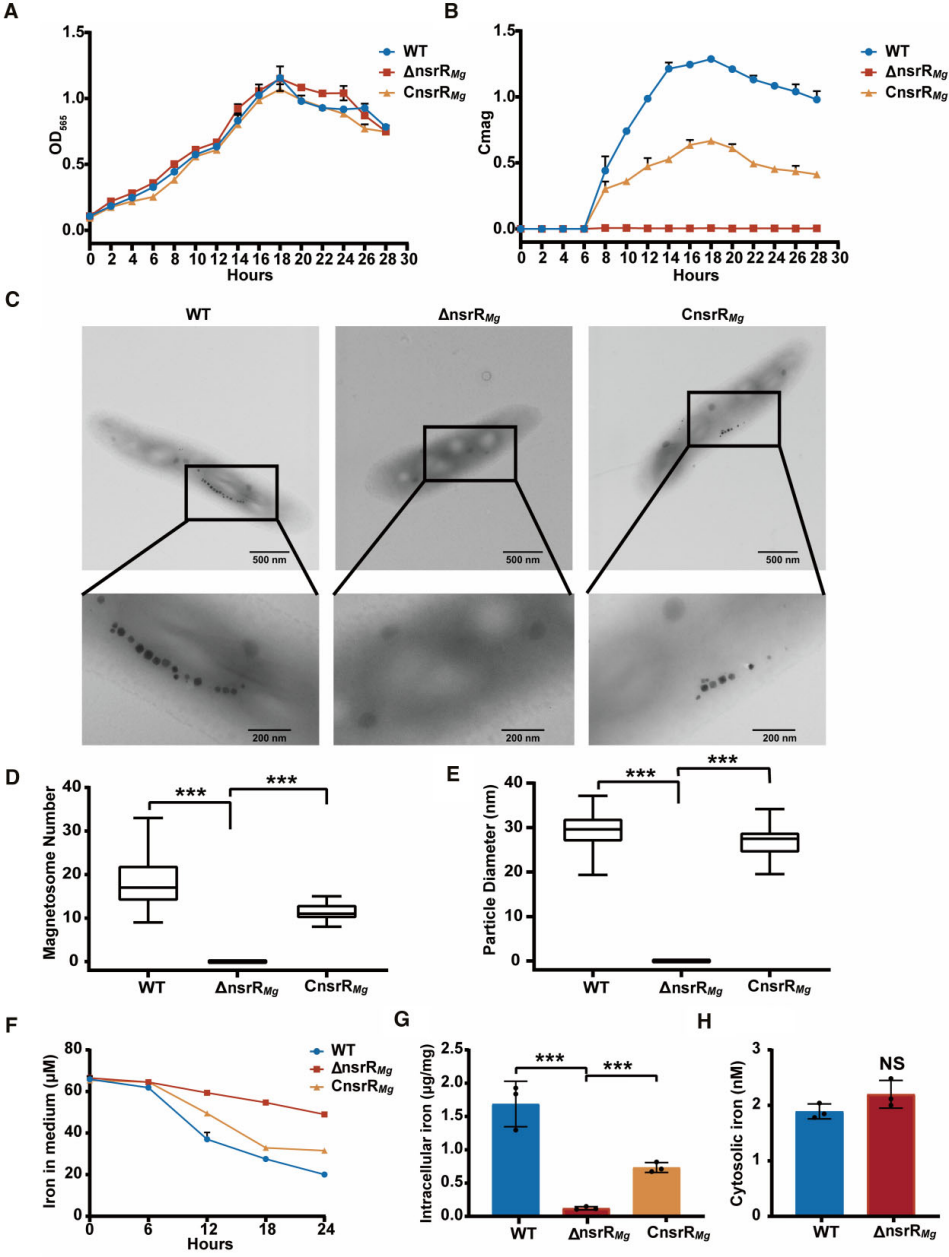
Phenotypic analysis of MSR-1 strains WT, ΔnsrR*_Mg_* and CnsrR*_Mg_*. (**A**) Growth curves for strains cultured in mSLM. Biomass is expressed as OD_565_. (**B**) Magnetic response (Cmag) curves. (**C**) TEM images with progressive magnification. Scale bars: 500 and 200 nm. (**D**) Box-plot charts of magnetosome numbers for WT, ΔnsrR*_Mg_* and CnsrR*_Mg_* (each *n* = 35). (**E**) Magnetosome sizes for WT (*n* = 643), ΔnsrR*_Mg_*(*n* = 0), and CnsrR*_Mg_*(*n* = 400). (**F**) Iron concentrations in medium at indicated time points during mSLM culture. (**G**) Intracellular iron content at 18 h. (**H**) Cytosolic iron content at 18 h. Statistical notations for this and subsequent figures: Error bars: mean ± SD from three biological replicates. *P< 0.05, **P< 0.01, ***P< 0.001, NS: no significant difference, based on unpaired two-tailed Student's *t*-test.

We constructed *nsrR_Mg_* in-frame deletion mutant ΔnsrR*_Mg_* (Supplementary Figure S1) and its complemented strain CnsrR*_Mg_*, in order to investigate the function of NsrR*_Mg_* in MSR-1. Cell growth (OD_565_) and magnetic response (Cmag value) analyses (57) were performed for ΔnsrR*_Mg_*, CnsrR*_Mg_* and WT strain. Growth patterns of ΔnsrR*_Mg_* and CnsrR*_Mg_* were similar to that of WT (Figure 1A). ΔnsrR*_Mg_* showed a striking loss of magnetic response, and Cmag value was partially complemented in CnsrR*_Mg_* (Figure 1B). TEM analysis of samples (35 cells) cultured for 24 h in mSLM (containing NH_4_Cl as nitrogen source) showed that there was no magnetite crystal in ΔnsrR*_Mg_* cells in contrast to revealed 18 ± 6 and 11 ± 2 magnetosomes per cell for WT and CnsrR*_Mg_*, respectively (Figure 1C and D). Mean magnetosome diameters were respectively 29.40 ± 3.79 (from 643 magnetosomes) and 26.81 ± 3.23 nm (from 400 magnetosomes) for WT and CnsrR*_Mg_* (Figure 1E). As nitrate was used as nitrogen source for MTB cultivation in some reports (14,32), we also analyzed the cells of WT, ΔnsrR*_Mg_* and CnsrR*_Mg_* cultured in nitrate medium by TEM, and the results were similar with those in mSLM (Supplementary Figure S3A-C). These findings indicate that NsrR*_Mg_* is necessary for magnetosome synthesis, but has no effect on cell growth at least under culture conditions we used.

Magnetosome synthesis depends on maintenance of redox environment. To investigate the effect of reactive oxygen species (ROS) on magnetosome formation and whether *nsrR_Mg_* deletion affects content of intracellular ROS, we measured intracellular ROS levels of WT, WT treated with 200 μM H_2_O_2_, and ΔnsrR*_Mg_* cultured in mSLM using fluorescent probe DCFH-DA. ROS levels in ΔnsrR*_Mg_* were higher than in WT, whereas lower than in H_2_O_2_-treated WT at four time points (6, 12, 18, 24 h) (Supplementary Figure S4A). TEM analysis showed that there were still fewer and smaller magnetite crystals in H_2_O_2_-treated WT than in WT, but no magnetite crystal in ΔnsrR*_Mg_* (Supplementary Figure S4B-D). These findings indicate that the complete loss of magnetosomes in ΔnsrR*_Mg_* was not due to the increased intracellular ROS level.

There are typically two possible direct reasons for absence of magnetosomes: (i) low availability of Fe^2+^ or Fe^3+^ ion; (ii) low expression level of magnetosome formation genes. Possibility (i) was evaluated by measuring iron absorption capability of the three strains. Iron absorption in WT occurred mainly between 6–18 h – the period during which cells rapidly absorb iron and form magnetosomes. Iron absorption was far lower for ΔnsrR*_Mg_* than for WT or CnsrR*_Mg_* (Figure 1F). Intracellular iron content after 18-h culture was 5-fold higher for WT than for ΔnsrR*_Mg_*(Figure 1G), whereas cytosolic iron content in these strains was similar (Figure 1H), indicating that lower iron content in ΔnsrR*_Mg_* was not due to iron scarcity in the cytosol, but rather to the absence of magnetosome biomineralization.

### NsrR*_Mg_* directly activates transcription of MGC genes

**Figure 2.**
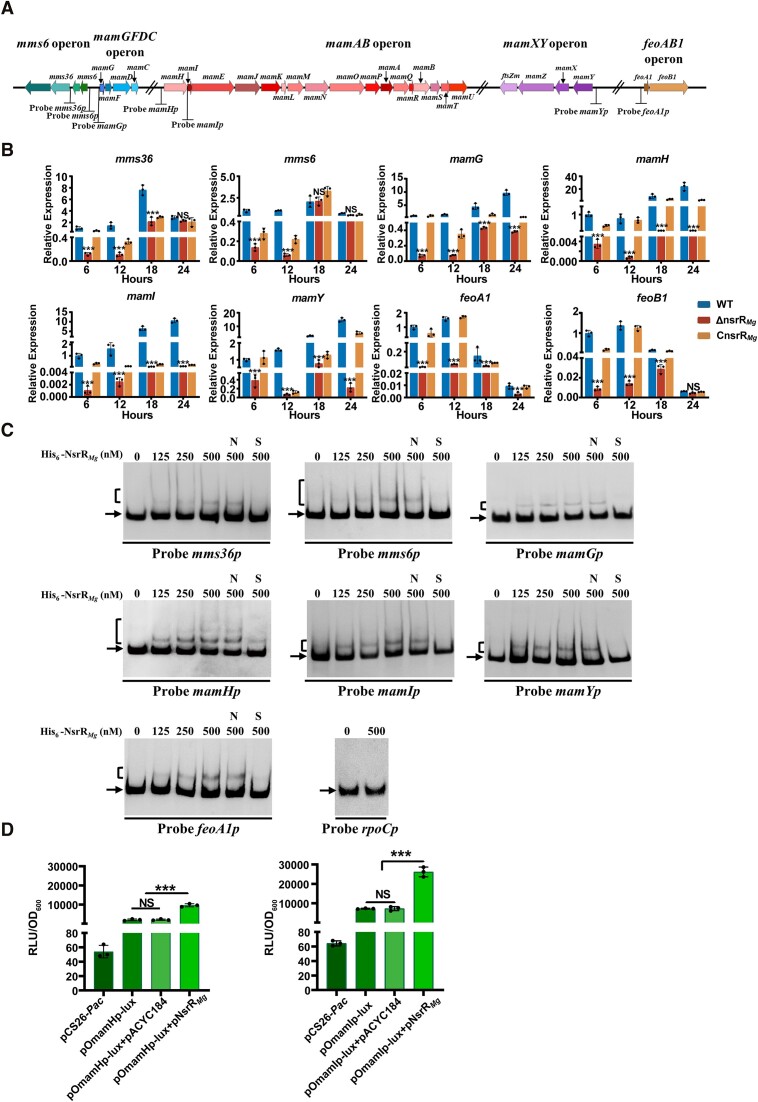
Direct activation of MGC genes by NsrR*_Mg_*. (**A**) Promoter probes for EMSAs (schematic). (**B**) qRT-PCR analysis of MGC genes in WT, ΔnsrR*_Mg_*, and CnsrR*_Mg_* cultured in mSLM. Reference gene: *rpoC*. Transcription level of each gene was expressed relative to that of WT at 6 h, defined as 1. (**C**) EMSAs of His_6_-NsrR*_Mg_* interactions with indicated promoter probes. Negative probe: *rpoCp*. Each lane contained 0.3 nM labeled probe. Lanes N and S: competition experiments using ∼400-fold unlabeled nonspecific probe *rpoCp* (N) or respective specific probe (S). Arrow: free probe. Bracket: NsrR*_Mg_*-DNA complex. (**D**) Effect of NsrR*_Mg_* on bioluminescence (values expressed as relative light units [RLU]) in *E. coli lux*-reporter system containing pOmamHp-lux (left) (or pOmamIp-lux, right) and pNsrR*_Mg_*. Plasmid controls: pCS26-*Pac* and pACYC184. Statistical notations as in Figure 1.

Effect of *nsrR_Mg_* deletion on expression of MGC genes was evaluated by qRT-PCR analysis. A 2016 review article summarized that MGC genes in MSR-1 are organized as five operons: *mms6*, *mamGFDC, mamAB*, *mamXY* and *feoAB1* (15) (Figure 2A). Among these, *mamAB* operon is most important for magnetosome formation, and *mamABEIKMPQ* in this operon are eight conserved genes for magnetosome biomineralization (20–22). D. Schüler's group reported that the promoter P(*mamH*) (hereafter referred to as *mamIp* to avoid confusion because it is in front of *mamI*) between *mamH* and *mamI* is the most important promoter in *mamAB* operon, and P*mms36* (i.e.*mms36p*) in *mms6* operon is also a key promoter (58). In *feoAB1* operon (which encodes the major iron transporters involved in magnetosome formation), *feoB1* gene is more important than *feoA1* (25). We accordingly performed qRT-PCR analysis of the first gene of each operon, and of individual genes of *mamABEIKMPQ*, *mms36*, and *feoB1*. WT, ΔnsrR*_Mg_* and CnsrR*_Mg_* were grown in mSLM for 6, 12, 18 or 24 h, and RNA samples were prepared. Transcription levels of *mamH* and eight conserved genes *mamABEIKMPQ* in *mamAB* operon were lower for ΔnsrR*_Mg_* than for WT at all four time points – in particular, it was ∼96.0- to 3278.8-fold lower at 12, 18 and 24 h (Figure 2B and Supplementary Figure S5). Levels of *mamG* (for *mamGFDC* operon), *mamY* (for *mamXY* operon), *mms36* and *mms6* (for *mms6* operon) were ∼0.3- to 23.8-fold lower in ΔnsrR*_Mg_* at two or four time points (Figure 2B). *feoA1* and *feoB1* expression levels were also low in ΔnsrR*_Mg_* (Figure 2B), consistent with iron absorption data (Figure 1F). Transcription levels of detected MGC genes were partially rescued in complemented strain CnsrR*_Mg_*(Figure 2B and Supplementary Figure S5). Results of protein identification and quantification revealed that levels of magnetosome-associated proteins encoded by MGC genes were dramatically lower for ΔnsrR*_Mg_* than for WT (Supplementary Figure S6), consistent with qRT-PCR data. These findings indicate that NsrR*_Mg_* promotes magnetosome formation by activating transcription of MGC genes, particularly that of *mamAB* operon, which contains genes essential for the biomineralization process.

The possibility that NsrR*_Mg_* directly regulates MGC genes was evaluated by EMSAs using soluble His_6_-tagged NsrR*_Mg_* purified from *E. coli* and promoter regions of MGC genes. Seven promoter probes (*mms36p*, *mms6p*, *mamGp*, *mamHp*, *mamIp*, *mamYp*, *feoA1p*) were designed and applied in EMSAs (Figure 2A), with probe *rpoCp*, corresponding to promoter region of *rpoC* (encodes RNA polymerase β subunit) as negative control. His_6_-NsrR*_Mg_* formed complexes with *mms36p*, *mms6p*, *mamGp*, *mamHp*, *mamIp*, *mamYp*, and *feoA1p*, but did not bind to *rpoCp* (Figure 2C). Binding specificity was evaluated by competition assays using ∼400-fold excesses of (i) unlabeled nonspecific probe *rpoCp*, which had no effect on retarded bands (lanes N), and (ii) unlabeled specific probes, which competed strongly with corresponding labeled probes for binding to NsrR*_Mg_* (lanes S) (Figure 2C). These results indicate that NsrR*_Mg_* regulates magnetosome formation directly through binding to all important promoter regions of MGC genes.

*In vivo* binding of NsrR*_Mg_* to above seven target promoters of MGC genes was confirmed by chromatin immunoprecipitation-quantitative PCR (ChIP-qPCR) experiments. Samples were taken from WT and ΔnsrR*_Mg_* grown in mSLM for various durations. Anti-NsrR*_Mg_* antibody was used to detect binding of NsrR*_Mg_* to its target promoters. No enrichment of NsrR*_Mg_* on *rpoCp* was detected. Enrichment levels of NsrR*_Mg_* on seven target promoters were higher for WT than for ΔnsrR*_Mg_* in all samples immunoprecipitated at various time points, and the strongest binding to each target promoter was observed at 18 h (Supplementary Figure S7). These findings indicate dynamic binding of NsrR*_Mg_* to these target promoters *in vivo*.

NsrR protein usually acts as a repressor (1,43). We used a *lux*-reporter system in *E. coli* (54) to further examine the regulatory relationship of NsrR*_Mg_* with *mamHp* and *mamIp* for core operon *mamAB*, and to confirm our finding that NsrR*_Mg_* acts as an activator of MGC genes. Three plasmids were constructed for this system: pNsrR*_Mg_* (based on pACYC184) for expression of NsrR*_Mg_*, pOmamHp*-*lux (based on pCS26-*Pac* bearing promoterless *lux* operon) and pOmamIp-lux for expression of *mamHp*- and *mamIp*-controlled *lux* operon. Both pOmamHp-lux and pOmamIp-lux gave higher level of bioluminescence relative to control plasmid pCS26-*Pac*, which gave only background level (Figure 2D), indicating that promoters *mamHp* and *mamIp* are recognized by *E. coli* RNA polymerase. Bioluminescence of transformant bearing pOmamHp-lux or pOmamIp-lux was much more strongly enhanced by pNsrR*_Mg_* than by control plasmid pACYC184 (Figure 2D). These findings demonstrate that NsrR*_Mg_* directly activates transcription of (*i.e*. enhances activity of) *mamHp* and *mamIp*.

### Determination of NsrR*_Mg_*-binding sites on *mamHp*

**Figure 3.**
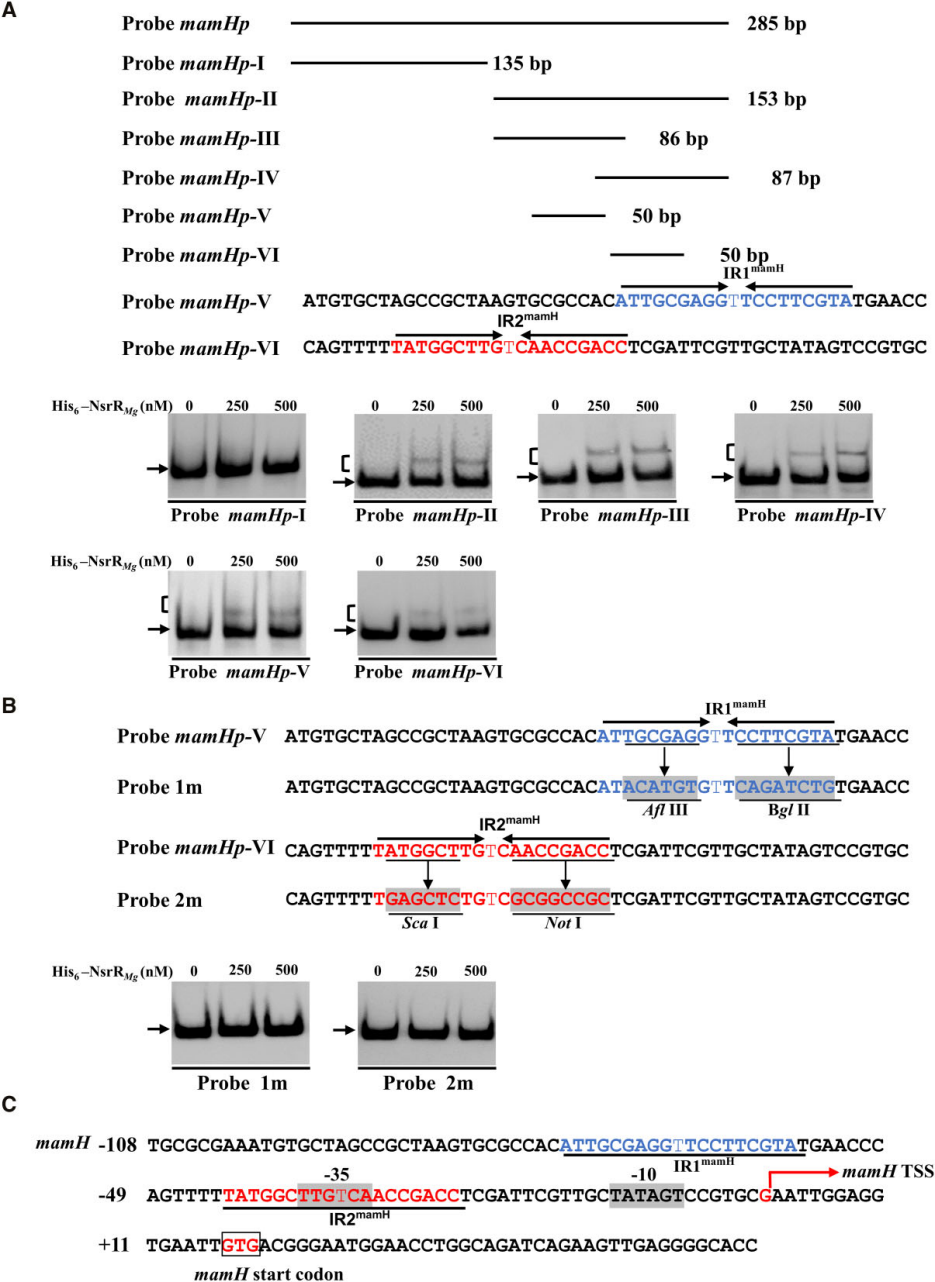
NsrR*_Mg_*-binding sites in *mamH* promoter region. (**A**) EMSAs of His_6_-NsrR*_Mg_* interactions with probes located within *mamH* promoter region. Relative probe positions and lengths are shown schematically. Each lane contained 0.3 nM labeled probe. Straight arrows: inverted direct repeats. (**B**) EMSAs using mutated 50-bp probes (1m, 2m) of *mamHp-*V and *mamHp-*VI. Each lane contained 0.3 nM labeled probe. Underlining: altered nucleotides. (**C**) Nucleotide sequences of *mamH* promoter region and NsrR*_Mg_*-binding sites. Numbers: distance (nt) from *mamH* TSS. Red bent arrow: *mamH* TSS. Shading: putative –10 and –35 regions. Box: *mamH* TSC. Underlining: NsrR*_Mg_*-binding sites.

Identification of precise NsrR*_Mg_*-binding sites is essential for understanding the regulatory mechanism of NsrR*_Mg_* on its target promoters. Many such attempts have been made using DNase I footprinting assays, but it was not possible to detect binding sites on promoter regions of target genes, most likely because of low DNA-binding activity of purified His_6_-NsrR*_Mg_*. As an alternative approach, we performed EMSAs using a series of overlapping probes to determine protected site(s) of NsrR*_Mg_* on *mamHp*, the first promoter of the core operon *mamAB*. The 285-bp *mamHp* probe was divided into two probes with only 3-bp overlap: *mamHp*-I (135-bp) and *mamHp-*II (153-bp) (Figure 3A). His_6_-NsrR*_Mg_* bound to *mamHp-*II but not to *mamHp-*I, indicating that the NsrR*_Mg_*-binding site(s) are located within the *mamHp-*II region. Next, *mamHp-*II was divided into two probes with 20-bp overlap: *mamHp-*III (86-bp) and *mamHp-*IV (87-bp) (Figure 3A). His_6_-NsrR*_Mg_* bound to both *mamHp-*III and *mamHp-*IV, indicating that they have at least two NsrR*_Mg_*-binding sites.

NsrRs reported so far generally form symmetric dimers and bind to imperfect palindromic sequences (43). DNAMAN analysis revealed that *mamHp-*III contains a 19-bp sequence (5′-ATTGCGAGGTTCCTTCGTA-3′, termed IR1^mamH^) similar to the consensus NsrR recognition motif (5′-VDHDYAWWWHWDWWRYRHB-3′) (V = A/C/G, D = A/G/T, H = A/C/T, Y = C/T, W = A/T, R = A/G, B = G/C/T) for γ-proteobacteria, and *mamHp-*IV contains a 19-bp sequence (5′-TATGGCTTGTCAACCGACC-3′, termed IR2^mamH^) similar to the consensus NsrR recognition motif (5′-BWWDYATHHNRRATVYHDN-3′) (N = A/T/C/G) for *Bacillus* and *Streptomyces* (43). IR1^mamH^ and IR2^mamH^ are not located within the 20-bp overlapping region. We constructed two 50-bp probes (without overlap), *mamHp-*V (within *mamHp-*III, containing IR1^mamH^) and *mamHp-*VI (within *mamHp-*IV, containing IR2^mamH^), to shorten the NsrR*_Mg_*-binding region (Figure 3A). Binding of His_6_-NsrR*_Mg_* to both *mamHp-*V and *mamHp-*VI was revealed by EMSAs, suggesting that sequences IR1^mamH^ and IR2^mamH^ both serve as target sites for NsrR*_Mg_* binding (Figure 3A). To further clarify the roles of sequences IR1^mamH^ and IR2^mamH^ in NsrR*_Mg_* binding, we generated two mutated probes by introducing mutations into the sequences: 1m (from *mamHp-*V) and 2m (from *mamHp-*VI) (Figure 3B). No binding of His_6_-NsrR*_Mg_* to 1m or 2m was observed under the same EMSA condition as for *mamHp-*V and *mamHp-*VI (Figure 3B). These findings indicate that two NsrR*_Mg_*-binding sites (IR1^mamH^, IR2^mamH^) are present on *mamH* promoter region, and that both are essential for NsrR*_Mg_* binding.

To clarify the mechanism whereby NsrR*_Mg_* regulates target *mamHp* for *mamAB* operon, we mapped by 5′RACE the transcriptional start site (TSS) of *mamH* to G, 16 nt upstream of *mamH* translational start codon (TSC) (Figure 3C and Supplementary Figure S8). NsrR*_Mg_*-binding site IR1^mamH^ extends from positions -57 to -75 relative to *mamH* TSS, and site IR2^mamH^ overlaps the putative -35 region (Figure 3C). Although the NsrR_*Mg*_-binding site on *mamHp* is unusual for an activator, it is analogous to previous reports that BldD binding overlaps the putative -35 region on *dptR3p* (59) and the TSS and -10 region on *eryBVIp* (60), and that BldD also activates*dptR3* and *eryBVI*. The mechanism of such transcriptional activation remains to be clarified. It is possible that NsrR*_Mg_* activates *mamAB* operon by either stabilizing RNA polymerase or promoting recruitment of the polymerase to *mamH* promoter.

### NsrR*_Mg_*responds to NO in regulation of magnetosome synthesis

**Figure 4.**
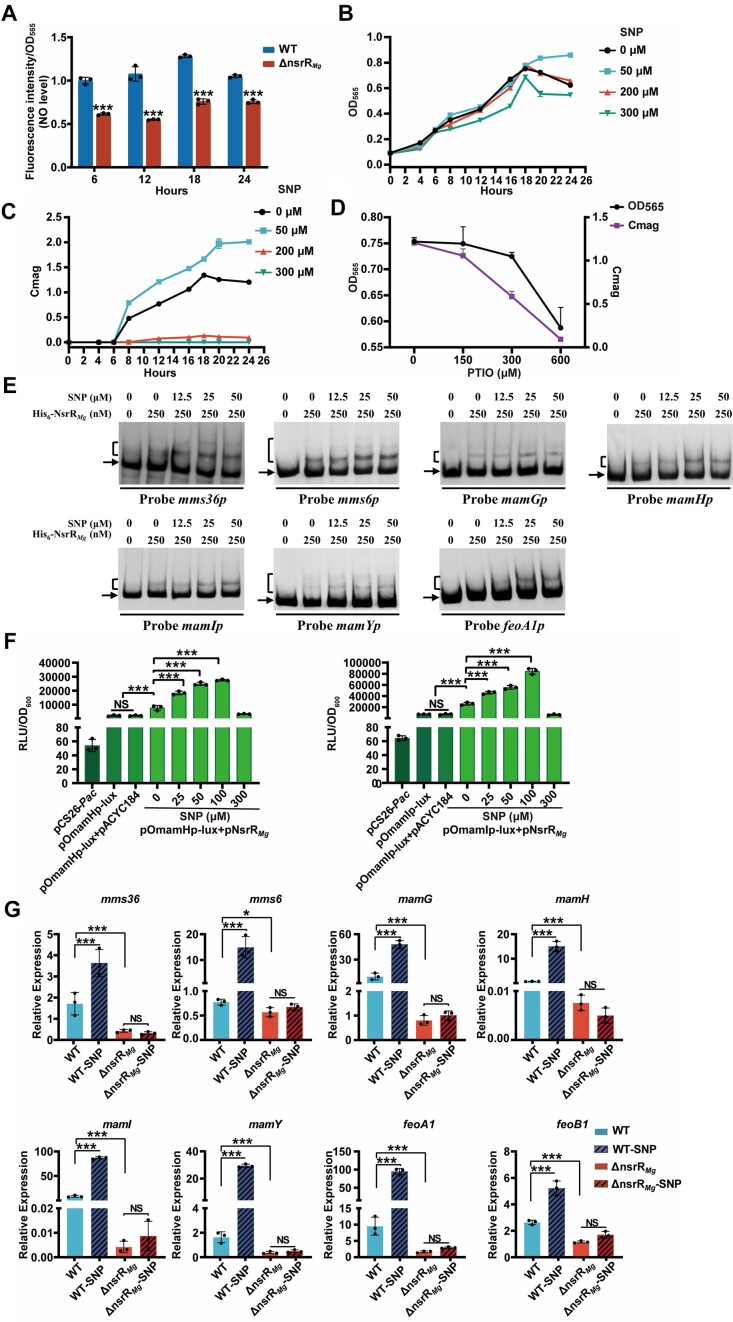
Effects of NO on cell growth, magnetosome formation, and transcription of MGC genes.**(A)** Intracellular NO levels in WT and ΔnsrR*_Mg_*. NO levels are expressed as relative fluorescence intensity. **(B, C)** Growth (**B**) and Cmag curves**(C)** of WT treated with indicated SNP concentrations. (**D**) Growth (OD_565_) and Cmag of WT cultured for 24 h in mSLM with indicated PTIO concentrations. (**E**) EMSAs of His_6_-NsrR*_Mg_* (250 nM) interactions with SNP at indicated concentrations. Each lane contained 0.3 nM labeled probe. (**F**) NO interactions with NsrR*_Mg_* determined with *E. coli* lux-reporter system containing pOmamHp-lux (or pOmamIp-lux) and pNsrR_*Mg*_. SNP at indicated concentrations was added to cultures. (**G**) qRT-PCR analysis of MGC genes in WT and ΔnsrR*_Mg_* cultured for 18 h in mSLM with or without SNP (50 μM). Statistical notations as in Figure 1.

The above experiments showed that NsrR*_Mg_* regulates magnetosome formation, but did not demonstrate whether NO is involved in the process. NO is often an intermediate metabolite of denitrification in Gram-negative bacteria. However, the nitrogen source in mSLM used for MSR-1 growth and magnetosome formation is ammonium chloride (NH_4_Cl) rather than nitrate or nitrite for denitrification. We therefore evaluated possible NO production during MSR-1 growth in mSLM, using DAF-FM DA fluorescent probe (61) to measure intracellular NO level. NO level was higher in WT than in ΔnsrR_*Mg*_, with maximum at 18 h (Figure 4A). Consistently, WT also showed maximal Cmag value at 18 h (Figure 1B).

To investigate effects of NO on cell growth and magnetosome formation, we added various concentrations of SNP (NO donor) and 2-(4-carboxyphenyl)-4,4,5,5-tetramethylimidazoline-1-oxyl 3-oxide (PTIO; NO scavenger) to mSLM prior to MSR-1 inoculation. During the period 0–18 h, MSR-1 growth (OD_565_) was unaffected by either 50 or 200 μM SNP treatment. After 18 h, growth ceased for 200 μM SNP-treated cells and control cells (no SNP), but continued for 50 μM SNP-treated cells. Growth of 300 μM SNP-treated cells was much less than that of control cells (Figure 4B). Cmag value was almost abolished by 200 or 300 μM SNP treatment, but was increased by 50 μM SNP treatment (Figure 4C). Cmag was lower for PTIO (150, 300, 600 μM)-treated cells than for control cells, and growth was strongly inhibited by 600 μM PTIO (Figure 4D). The above findings, taken together, suggest that a defined range of NO level (not exceeding a certain limit) is necessary for magnetosome formation. This is reasonable in view of the cytotoxic and growth-inhibitory effects of high NO levels.

We performed EMSAs with SNP concentration gradients (≤50 μM) to examine the effect of NO on NsrR*_Mg_* DNA-binding activity. In the presence of SNP, NsrR_*Mg*_ affinity for target MGC promoters was enhanced (Figure 4E), indicating that NsrR*_Mg_* responds to NO signaling in regulation of target MGC genes. For *in vivo* confirmation, we added SNP to *E. coli* lux-reporter system containing pOmamHp-lux (or pOmamIp-lux) and pNsrR_*Mg*_. Bioluminescence was increased in dose-dependent manner at low SNP concentrations (25–100 μM), but reduced by high SNP concentration (300 μM), presumably as a result of cytotoxicity (Figure 4F), indicating that NsrR*_Mg_* senses NO level for precise regulation of target gene expression. This concept was evaluated by measuring transcription of MGC genes in 50 μM SNP-treated WT and ΔnsrR*_Mg_*, with total RNAs isolated after 18 h treatment. Transcription levels of MGC genes (*mms36*, *mms6*, *mamG*, *mamH*, *mamI*, *mamY*, *feoA1*, *feoB1*) after such treatment were increased ∼1- to 2-fold in WT, but unaffected in ΔnsrR*_Mg_* (Figure 4G), indicating that appropriate NO level promotes magnetosome formation by activating NsrR*_Mg_*-mediated transcription of MGC genes. The above findings, taken together, demonstrate that NsrR*_Mg_* utilizes NO as an effector to modulate DNA-binding activity, target MGC gene expression, and consequent magnetosome formation.

### Confirmation of nitrification-denitrification metabolic pathway in MSR-1

Intracellular NO is detectable during growth of MSR-1 in mSLM containing NH_4_Cl as nitrogen source; however, the metabolic pathway for NO production is unclear. A particular group of heterotrophic bacteria has been shown to perform simultaneous nitrification and denitrification under aerobic or microaerobic conditions (62,63). During nitrification, ammonium (NH_4_^+^) is first converted to nitrite (NO_2_^−^), catalyzed by the enzymes ammonia monooxygenase (AMO) and hydroxylamine oxidoreductase (HAO), and then transformed to nitrate (NO_3_^−^) by nitrite oxidoreductase (NXR). The dissimilatory denitrification pathway consists of four enzymatic steps for serial reduction of NO_3_^−^ → NO_2_^−^ → NO → nitrous oxide (N_2_O) → nitrogen (N_2_) by periplasmic nitrate reductase (NAP), nitrite reductase (NIR), nitric oxide reductase (NOR), and nitrous oxide reductase (NOS), respectively. MSR-1 genome contains genes for the two pathways: *amoA* [*MGMSRv2_3360*, 46% and 28% amino acid identity with its homolog in *Skermanella stibiiresistens* (EWY39946) and *Leptospirillum ferrodiazotrophum* (EES52284)], *haoA* [*MGMSRv2_3858*, 28% identity with its homolog in *Desulfobacteraceae bacterium* (VEN74915)] and *nxrAB* (*MGMSRv2_0154* and *MGMSRv2_0155*, 32% and 44% identities with their homologs in *Nitrobacter* and *Nitrospira*, respectively) for nitrification; *napFDAGHBC* (*MGMSRv2_2000* to *MGMSRv2_2006*), *nirTQ* (*MGMSRv2_1402*, *MGMSRv2_1401*), *nirS* (*MGMSRv2_1403*), *norCBQD* (*MGMSRv2_1714* to*MGMSRv2_1717*), and *nosZ* (*MGMSRv2_1430*) for denitrification (33). Denitrification is the only possible pathway to form NO, and no other pathway genes (such as nitric oxide synthase gene) for NO production were found in MSR-1. Thus, MSR-1 can presumably oxidize NH_4_^+^ to NO_2_^−^ or NO_3_^−^ through nitrification, and then denitrify these products to intermediate NO through denitrification.

The above hypothesis was tested by a series of ^15^N isotope tracer experiments. When WT and ΔnsrR_*Mg*_ were cultured for 18 h in mSLM with isotopically labeled ^15^NH_4_Cl (10% ^15^N), small amounts of ^15^N-NO_2_^−^ and ^15^N-NO_3_^−^ were produced in both strains: 2377.18‰ δ^15^N of NO_2_^−^ and 76073.71‰ δ^15^N of NO_3_^−^ in WT; 665.51‰ δ^15^N of NO_2_^−^and 1359.68‰ δ^15^N of NO_3_^−^ in ΔnsrR_*Mg*_ (Supplementary Figure S9A). These findings demonstrate that MSR-1 can produce nitrite and nitrate from NH_4_Cl through nitrification. Nitrite levels in mSLM at 18 h were measured indirectly using Griess reagent. Nitrite level was higher for WT than for ΔnsrR_*Mg*_ culture (Supplementary Figure S9B), consistent with endogenous nitrite levels in the two strains. No nitrite was detected in control medium (no growth of strains), demonstrating that nitrite production resulted from MSR-1 metabolism, not other factors. When a widely used nitrification inhibitor, 3,4-dimethylpyrazole phosphate (DMPP) (64), was added to mSLM, MSR-1 growth and magnetosome production were strongly inhibited by 1000 μM treatment (Supplementary Figure S10A-D), confirming the existence of nitrification pathway in MSR-1.

We attempted to confirm the existence of denitrification pathway by detecting N_2_, the end product of denitrification. N_2_ was below detectable level when ^15^NH_4_Cl was used as sole nitrogen source, and we therefore added Na^15^NO_3_ to ^15^NH_4_Cl-containing mSLM. After 18 h growth, WT and ΔnsrR_*Mg*_ produced 12.69‰ and 18.97‰ δ^15^N of N_2_, respectively (Supplementary Figure S9C), demonstrating the existence of denitrification pathway in MSR-1 under our culture conditions, and confirming that MSR-1 can produce endogenous NO from NH_4_Cl via nitrification-denitrification pathway.

### NsrR_*Mg*_ directly regulates nitrification and denitrification genes in response to NO

**Figure 5.**
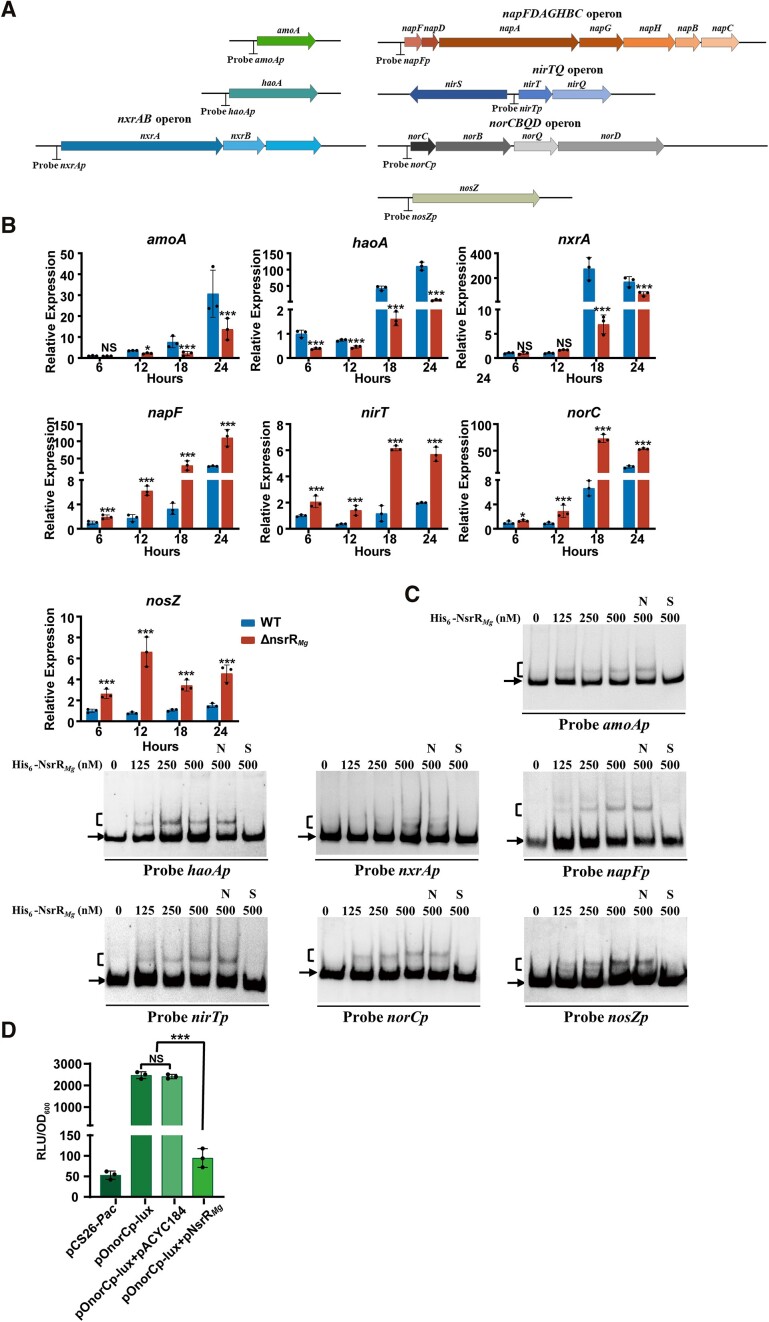
Direct regulation of nitrification and denitrification genes by NsrR*_Mg_*. (**A**) Gene organizations and promoter probes for EMSAs (schematic). (**B**) qRT-PCR analysis of nitrification and denitrification genes in WT and ΔnsrR*_Mg_* grown in mSLM. Reference gene: *rpoC*. Transcription level of each gene was expressed relative to that of WT at 6 h, defined as 1. (**C**) EMSAs of His_6_-NsrR*_Mg_* interactions with indicated promoter probes. Notations as in Figure 2C. (**D**) Effect of NsrR*_Mg_* on bioluminescence in *E. coli lux-*reporter system containing pOnorCp-lux and pNsrR*_Mg_*. Statistical notations as in Figure 1.

Involvement of NsrR_*Mg*_ in regulation of NO production was suggested by the lower NO level in ΔnsrR_*Mg*_ than in WT. We examined this possibility by applying qRT-PCR to assess expression of genes involved in nitrification and denitrification, using the same RNA preparations described in Figure 2B. Transcription levels of three nitrification genes (*amoA*, *haoA*, *nxrA*) were lower in ΔnsrR_*Mg*_ than in WT, whereas levels of four denitrification genes (*napF*, *nirT*, *norC*, *nosZ*) were higher in ΔnsrR_*Mg*_ (Figure 5B), consistent with NO levels in the strains (Figure 4A). These findings suggest that NsrR*_Mg_* acts as an activator of nitrification genes, but as a repressor of denitrification genes.

Possible direct regulation of the above genes by NsrR*_Mg_* was evaluated by EMSAs using probes *amoAp*, *haoAp*, *nxrAp* (for *nxrAB* operon), *napFp* (for *napFDAGHBC* operon), *nirTp* (for *nirTQ* operon), *norCp* (for *norCBQD* operon), and *nosZp* (Figure 5A). His_6_-NsrR*_Mg_* bound specifically to each of the promoter probes, indicating direct regulation of these genes and their corresponding operons by NsrR*_Mg_* (Figure 5C). Direct binding of NsrR*_Mg_* to above seven target promoters of nitrification and denitrification genes *in vivo* was further confirmed by ChIP-qPCR assays (Supplementary Figure S11).

We evaluated the possibility that NsrR*_Mg_* also acts as a repressor of denitrification genes by examining its regulatory relationship with target *norCp* in the *E. coli lux*-reporter system. Expression plasmid pNsrR*_Mg_* strongly reduced bioluminescence of transformant bearing pOnorCp-lux (Figure 5D), indicating that NsrR*_Mg_* directly represses *norCp* transcription.

**Figure 6.**
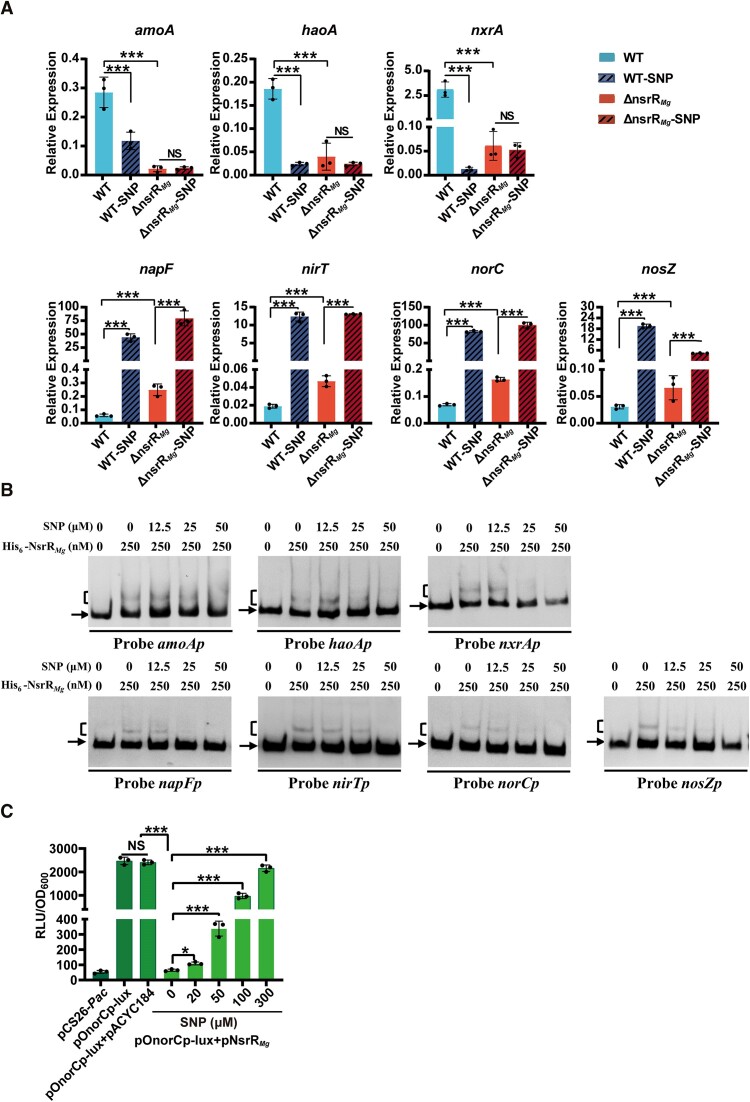
Effect of NO on expression of nitrification and denitrification genes. (**A**) qRT-PCR analysis of nitrification and denitrification genes in WT and ΔnsrR*_Mg_* grown in mSLM with or without SNP (50 μM) for 18 h. (**B**) EMSAs of His_6_-NsrR*_Mg_* (250 nM) interactions with SNP at indicated concentrations. (**C**) NO interactions with NsrR*_Mg_* in *E. coli lux-*reporter system containing pOnorCp-lux and pNsrR*_Mg_* with SNP added at indicated concentrations. Statistical notations as in Figure 1.

qRT-PCR was applied to examine effect of NO on expression of nitrification and denitrification genes, using the same RNA samples described in Figure 4G. Treatment with 50 μM SNP notably reduced transcription levels of nitrification genes *amoA*, *haoA* and *nxrA* in WT, but had no such effect in ΔnsrR*_Mg_*, indicating that these genes are repressed by NO under the control of NsrR*_Mg_*. In contrast, transcription levels of denitrification genes *napF*, *nirT*, *norC* and *nosZ* were increased ∼100-fold by 50 μM SNP treatment in both WT and ΔnsrR*_Mg_* (Figure 6A), indicating that NO strongly derepressed expression of these genes. These four denitrification genes were all targeted by NsrR*_Mg_*; therefore, the finding that SNP treatment enhanced their expression in ΔnsrR*_Mg_* suggests that they are derepressed by NO in both NsrR*_Mg_*-dependent and NsrR*_Mg_*-independent manners (i.e. presumably depending on other NO sensor(s) not identified here).

Results of EMSAs using SNP indicated that binding strength of His_6_-NsrR*_Mg_* to probes *amoAp*, *haoAp*, *nxrAp*, *napFp*, *nirTp*, *norCp* and *nosZp* was inversely correlated with SNP concentration (≤50 μM) (Figure 6B), and that NO acts as an effector of NsrR*_Mg_*, reducing its affinity for those seven promoter regions. In the *E. coli lux*-reporter system, bioluminescence level in transformant containing pOnorCp-lux and pNsrR*_Mg_* gradually increased in association with SNP concentration increase (Figure 6C), confirming that NO is involved in derepression of *norCp* by NsrR*_Mg_*.

Proteins containing Fe–S cluster are usually O_2_-sensitive (65). To investigate whether anaerobically purified His_6_-NsrR*_Mg_* (Supplementary Figure S12A) contains Fe–S cluster, we performed the UV-visible absorbance spectrum. The purified His_6_-NsrR*_Mg_* had absorption peaks around 412 and 460 nm, which are characteristics of [4Fe–4S] and [2Fe–2S] clusters (66), respectively, suggesting the presence of a mixture of [4Fe–4S] and [2Fe–2S] clusters. The two characteristic peaks disappeared completely after 4 h of air exposure (Supplementary Figure S12B), indicating that the Fe–S cluster of NsrR*_Mg_* is very sensitive to O_2_. Possible effect of O_2_ on NsrR*_Mg_* DNA-binding activity was investigated by EMSAs using O_2_ donor SPC (≤200 μM). Anaerobically purified His_6_-NsrR*_Mg_* generated shifted bands with target promoters, whereas retarded signals declined or disappeared with either increased His_6_-NsrR*_Mg_* exposure time to air (1–4 h), or increased SPC concentration (Supplementary Figure S13). These findings demonstrate that NsrR*_Mg_* also acts as an O_2_ sensor, thus ensuring expression of target genes under appropriate O_2_ concentration.

## Discussion

Calcium-based biomineralization widely occurs in animal skeleton formation and development. The well-known example is the calcium phosphate composition of vertebrate bones and teeth. A rare exception among animals is a deep-sea snail (*Chrysomallon squamiferum*), the only metazoan that possesses an iron sulfide shell (67). However, a wide variety of bacteria are capable of accumulating minerals intracellularly (68). In view of the diversity of these bacteria and the minerals they accumulate (which include iron, cadmium, selenium, silver, nickel, uranium, and calcium carbonate), studies focused on them are likely to elucidate the origins and basic mechanisms of biomineralization processes in higher organisms (68). MTB appeared early in evolution and are a useful model for studies of prokaryotic biomineralization (47). However, direct regulators of *mamAB* operon essential for biomineralization in MTB have been unknown until now. The present findings demonstrate that NsrR*_Mg_*, the NO sensor in *M. gryphiswaldense* strain MSR-1, is the direct regulator of *mamAB* operon and other operons within MGC, and that NsrR*_Mg_* and its responsive signaling molecule NO play key roles in regulation of magnetosome formation. Although NsrR homologs are found in a wide variety of bacteria from diverse ecological niches, they are not in all non-magnetic bacteria. However, NsrR homologs are present in all genome-annotated MTB, implying their conserved function as a regulator of magnetosome biomineralization.

These findings seem surprising, because the magnetotactic nature of MTB is generally viewed as flagellum-based aerotaxis with the aid of geomagnetic field (28). Why would MTB utilize a NO sensor, rather than an O_2_ sensor, as the main regulator of biomineralization processes? A 2017 study by Y. Pan's group suggests a possible explanation (11). The process of magnetosome formation was evidently well established prior to the Great Oxygenation Event (GOE) in the Paleoproterozoic era (2.4 billion years [Gyr] ago). O_2_ content in the early ocean was negligible prior to GOE (69), and there was accordingly no purpose for MTB to evolve an O_2_ sensor. During that time, NO could be produced by lightning strikes from CO_2_ and N_2_ in Earth's atmosphere. Thus, NO gradually accumulated throughout the Hadean (4.5–3.8 Gyr ago) and Archean (3.8–2.5 Gyr ago) eons (70). Photochemical reactions involving NO and water vapor generated various acids (*e.g*. HNO, HNO_2_, HNO_3_, HO_2_NO_2_) that were transferred from the atmosphere to the ocean by rain (71). Levels of solar UV radiation impacting the surface of the Archean ocean were orders of magnitude higher than today (72), and NO_2_^−^ was readily converted to NO by UV radiation (73,74) (Supplementary Figure S14A-C). It has been proposed that NO sensors were present and evolving during the Archean (75). Some comparative studies even suggest that NO coupled regulatory systems are as old as cellular organization *per se*, and originated around the beginning of biological evolution, ∼3.8–3.5 Gyr ago (76). These considerations are consistent with utilization of NsrR by MTB as a primary sensor for regulating biomineralization processes.

Another point should be addressed: if O_2_ was essentially absent in the atmosphere and ocean, how were biomineralization processes advantageous to ancient prokaryotes? Along this line, we hereby propose a hypothesis, described below, regarding the significance of magnetosomes in Archean oceans and in evolutionary events since that time.

Nitrogen is an essential nutrient for all life on Earth. Because nitrogen was present mainly in the atmosphere (77), it was necessary for Archean organisms to move near the ocean surface to perform nitrogen fixation for nutritional purposes. Studies by several groups suggest that the latest ‘universal common ancestor’ of all cells was capable of nitrogen fixation (78), and that this process was developed prior to GOE (79,80). On the other hand, NO and NO_2_^−^ were also present at higher concentrations near the ocean surface (as described above), and were potentially toxic to microorganisms. Furthermore, solar UV radiation was intense at the Archean ocean surface (72). Prokaryotes, in order to survive, needed to develop mechanisms to avoid these dangers. MTB evolved a ‘toolkit’ for formation of magnetosomes, which facilitated downward orientation and more efficient swimming away from nitrosative stress and UV radiation (Supplementary Figure S14D-G). In addition, L.L. Moroz & A.B. Kohn proposed that NO and NO_2_^−^ functioned as acceptor molecules for the first biological denitrification pathways in the early Archean ocean (76). It is possible that predecessors of MTB utilized denitrification pathways for energy production, thus reducing intracellular NO and NO_2_^−^ levels, similarly to MSR-1 processes observed in the present study.

Continued, gradual increase of atmospheric O_2_ level subsequent to GOE did not eliminate the regulatory roles of NO and its sensor NsrR in the ‘new world’. NsrR still participates in newly developed systems and displays newly developed functions; *e.g*. it evolved as an O_2_ sensor based on its redox-active Fe–S cluster. NsrR*_Mg_* can sense alterations of O_2_ concentration, and loses its DNA-binding activity under aerobic or hyperoxic conditions whereby magnetosome biomineralization becomes impossible. Because NsrR*_Mg_* is O_2_-sensitive, its DNA-binding activity is low during the typical EMSA conditions. Our EMSA results showed that only a small fraction of DNA probes was bound to NsrR*_Mg_* and the competition experiments using unlabeled specific probe sometimes did not lead to disappearance of the NsrR*_Mg_*-DNA complex. An improved EMSA method under anaerobic condition would be developed in future studies to solve such problems.

**Figure 7.**
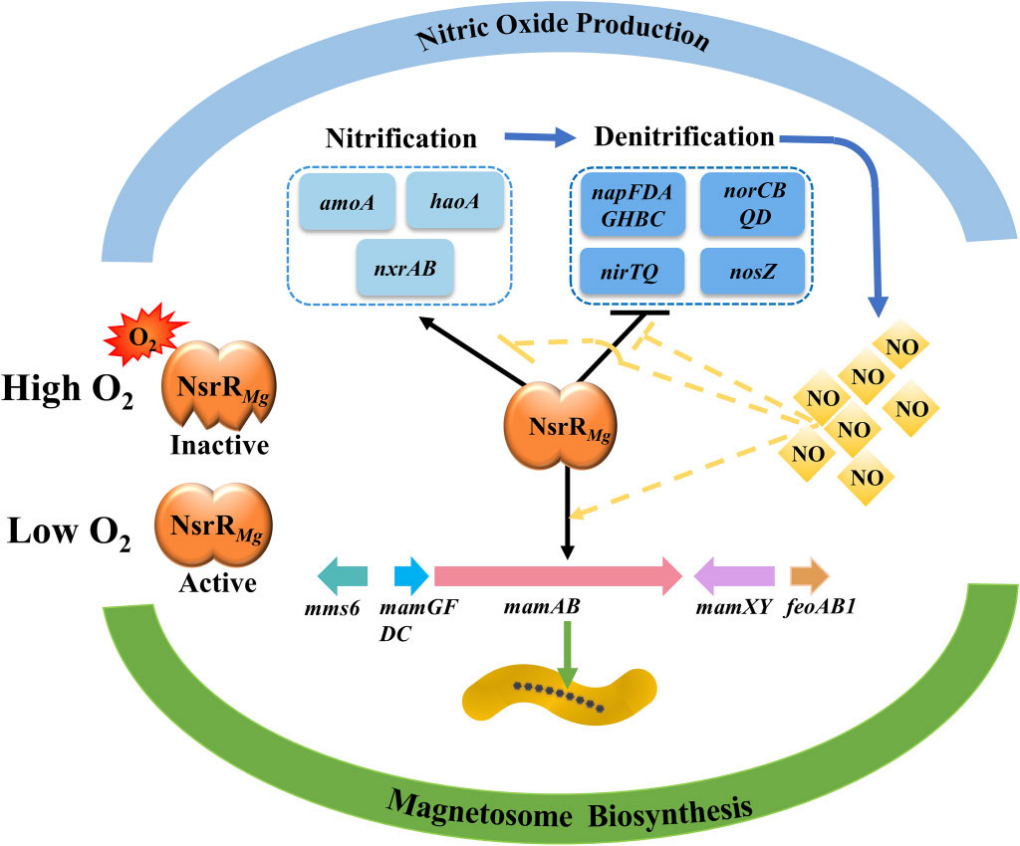
Proposed model of NsrR*_Mg_*-mediated regulation of magnetosome biosynthesis, nitrification, and denitrification genes in response to NO in MSR-1. Black solid-line arrow: activation. Black solid-line bar: repression. Yellow dashed-line arrow: enhancement by NO of DNA-binding activity of NsrR*_Mg_*. Yellow dashed-line bar: reduction by NO of DNA-binding activity of NsrR*_Mg_*. Green arrow: magnetosome biosynthesis. Blue arrow: NO production.

A proposed model for NsrR*_Mg_*-mediated regulation of magnetosome biosynthesis, nitrification, and denitrification genes in response to NO in MSR-1, based on present findings, is shown in Figure 7. Under high O_2_ concentration, NsrR*_Mg_* is inactive. Under low O_2_ concentration (hypoxic environment), NsrR*_Mg_* is activated and binds to promoter regions of MGC, nitrification, and denitrification genes. Endogenous NO is generated through nitrification-denitrification pathway. When it reaches a specific threshold level, it is sensed by NsrR*_Mg_* and changes DNA-binding activity of NsrR*_Mg_*, resulting in altered expression of the above target genes. Depending on the target, NO plays differing roles in modulation of DNA-binding ability of NsrR*_Mg_*. A certain amount of NO releases NsrR*_Mg_* from promoter regions of nitrification and denitrification genes, but enhances affinity of NsrR*_Mg_* for promoter regions of MGC genes; this results in increased expression of denitrification and MGC genes, but reduced expression of nitrification genes. Increased expression of MGC genes promotes magnetosome formation. High NO levels are cytotoxic, and inhibition of nitrification results in smaller amounts of nitrite and nitrate available for NO production. Enhancement of denitrification promotes conversion of NO to end product N_2_, resulting in appropriate NO concentration in cells.

The *E. coli* NsrR regulon includes at least 62 genes involved in NO stress response, NO metabolism, carbon and energy metabolism, stress responses, proteolysis, transport processes, motility and biofilm development (81). More target genes need to be identified in order to clarify broader roles of NsrR*_Mg_* in MSR-1. Analysis of NsrR*_Mg_*-binding promoter regions revealed presence of 19-bp IR1-like sequences in *mamHp*, *mamIp*, *mamGp*, *mamYp*, *mms6p*, *mms36p* and *feoA1p*. WebLogo (http://weblogo.berkeley.edu) analysis of these seven sequences generated a consensus sequence: 5′-WNYBBNWSNBDVSTTSSNN-3′ (W = A/T; S = C/G; Y = C/T; D = A/G/T; B = T/C/G; V = A/C/G; N = A/T/C/G (Supplementary Figure S15A). Each of the 14 NsrR*_Mg_* target promoter regions contains a 19-bp IR2-like sequence, and analysis of these sequences generated a second consensus sequence: 5′-NNNNNNWNVWVWWNNNHNN-3′ (H = A/C/T) (Supplementary Figure S15B). Scanning of MSR-1 genome by the tool PREDetector (82) using the two 19-bp consensus NsrR*_Mg_*-binding sequences led to prediction of > 200 putative NsrR*_Mg_* target genes (cut-off score ≥ 8.5), including well-annotated genes involved in nitrogen metabolism, iron metabolism, energy metabolism, or antioxidant function (Supplementary Table S3). Ongoing studies by our group will further elucidate the complex roles of NsrR and NO in MTB, by identifying additional NsrR*_Mg_* targets.

## Supplementary Material

gkad1230_Supplemental_File

## Data Availability

The data underlying this article are available in the article and in its online supplementary material.
