# Supplementary material for: Nitric oxide sensor NsrR is the key direct regulator of magnetosome formation and nitrogen metabolism in Magnetospirillum
Source: Nucleic Acids Res. 2024 Jan 9;52(6):2924–41. doi: 10.1093/nar/gkad1230 (PMC11014258; doi:10.1093/nar/gkad1230)
Supplement: gkad1230_Supplemental_File [file gkad1230_supplemental_file.pdf]

Supplementary information

Nitric oxide sensor NsrR is the key direct regulator of magnetosome formation and nitrogen metabolism in *Magnetospirillum*

Bo Pang, Haolan Zheng, Shijia Ma, Jiesheng Tian*, and Ying Wen*

State Key Laboratory of Animal Biotech Breeding and College of Biological Sciences, China Agricultural University, Beijing 100193, China.

***Corresponding authors:**

Jiesheng Tian; e-mail: tianhome@cau.edu.cn, phone: +86-10-62733751.

Ying Wen; e-mail: wen@cau.edu.cn, phone: +86-10-62732715.

SUPPLEMENTARY MATERIALS AND METHODS

Extracellular nitrite level

Nitrite content in MSR-1 culture media was determined using nitrite assay kit (Beyotime; China). MSR-1 strains were cultured in mSLM with 60 μM ferric citrate for 18 h, and centrifuged to obtain supernatant. Supernatant samples (50 μL) were reacted with equal volume of Griess reagent (Beyotime), and absorbance at 540 nm was obtained from microplate reader (SpectraMax Plus, BioTek; USA). Total nitrite concentration was calculated from a standard curve constructed using nitrate standards provided in the kit.

The release of NO in SNP solution incubated with intact or proteinase-treated magnetosomes for 1 h was determined by measuring nitrite content as mentioned above using total nitric oxide assay kit (Beyotime; China). To measure the capabilities of magnetosomes to convert NO to nitrate, nitrate reductase, NADPH, and flavin adenine dinucleotide (FAD) in the kit were added to reaction solution containing SNP and magnetosomes, and incubated for 30 min at 37°C. Then lactate dehydrogenase (LDH) was added to remove NADPH and OD_{540} was measured after reacting with Griess reagent.

NO_3^-, NO_2^-, and N_2 isotope detection

MSR-1 strains were inoculated into 200-mL serum bottles containing 100 mL mSLM with $^{15}NH_4Cl$ (10% ^{15}N atom; Macklin, China) (termed mSLM-1) or with $^{15}NH_4Cl$ and 1 mg/L $Na^{15}NO_3$ (10% ^{15}N atom; Macklin) (termed mSLM-2), and incubated in sealed culture for 18 h at 30°C with shaking (100 rpm). ^{15}N isotope ratios of $^{15}NO_3^-$ and $^{15}NO_2^-$ in bacterial liquid of samples cultured in mSLM-1, and of $^{15}N_2$ in headspace of samples cultured in mSLM-2, were measured at the Environmental Stable Isotope Laboratory of the Chinese Academy of Agricultural Sciences (AESIL, CAAS) by gas chromatography/isotope ratio mass spectrometry (GC-IRMS, Delta V Plus-Precon; Germany). Relative abundance of ^{15}N ($\delta^{15}N$) was calculated by the method of Toyoda and Yoshida (1).

Phylogenetic analysis of NsrR

Alignment of amino acid sequences of NsrR proteins was performed using MUSCLE algorithms (2) in MEGA (3), and poorly aligned portions were eliminated by Gblocks method (4). A phylogenetic tree was constructed using MEGA 10 (3) with maximum likelihood algorithm (5). Bootstrap values were calculated using 100 repetitions.

Rapid amplification of cDNA ends (5' RACE)

TSS of *mamH* was identified by 5' RACE (6,7). Total RNAs (4 μg) extracted from WT cultured in mSLM for 18 h were reverse transcribed using gene-specific primer mamH-sp1 (40 pmol). Purified cDNAs were treated with terminal transferase (TaKaRa; China) for addition of oligo(dA)-tail to 3′ ends. Tailed cDNAs were PCR-amplified with anchor primer oligo(dT) and specific nested primer mamH-sp2 to generate a single specific band. This band was purified for sequencing, and TSS was mapped as the first nucleotide following oligo(dA) sequence.

UV-visible absorption spectroscopy

UV-visible absorption spectra were obtained using a Lambda 35 UV/VIS spectrometer (PerkinElmer, USA). Effect of O_2 on the Fe-S cluster of $NsrR_{Mg}$ was investigated by recording the absorption spectra of anaerobically purified His_6-$NsrR_{Mg}$ at different time intervals up to 4 h.

Preparation of antibody against $NsrR_{Mg}$

An anti-$NsrR_{Mg}$ antibody was produced from the serum of a rabbit that had been immunized with anaerobically purified $NsrR_{Mg}$ protein by Beijing Protein Innovation (BPI; China).

Chromatin immunoprecipitation-quantitative PCR (ChIP-qPCR)

Cells of WT and $\Delta nsrR_{Mg}$ cultured in mSLM for various times were cross-linked by addition of 1% formaldehyde for 30 min. Then glycine (final concentration 125 mM)

was added to stop the cross-linking, and incubation was continued for 5 min at 100 rpm. The treated cells were harvested and processed as described previously to get input samples (positive control) in immunoprecipitation experiments (8).

One milliliter input sample containing 2 mg protein was used per immunoprecipitation, and 10 μL was kept as input DNA. After precleared with 50 μL Protein G Sepharose/salmon sperm DNA (GE Healthcare; USA), ChIP was performed using 2 μL anti-NsrR$_{Mg}$ antibody. Immunoprecipitated DNA was quantified by real-time PCR with primer pairs listed in Supplementary Table S2 and ΔnsrR$_{Mg}$ strain was used as negative control. ChIP-qPCR data were normalized by the input DNA and presented as a percentage of input DNA.

Protein identification and quantification

Cells of WT and ΔnsrR$_{Mg}$ cultured in mSLM for 12 h were harvested, ground with liquid nitrogen, resuspended in lysis buffer (50 mM NaH_2PO_4, 300 mM NaCl), and sonicated on ice to obtain extracted protein solution. After being quantified by the Bradford method, the proteins were digested with trypsin using a modified FASP method (9) to obtain peptides. Nanospray ESI-MS was performed on a Thermo Q-Exactive high resolution mass spectrometer (Thermo Scientific, Waltham, MA, USA) to analyze peptide samples. Raw data from the mass spectrometer were preprocessed by PeaksOnline (PeaksOnline X build 1.8) for peak picking, and the acquired peptide sequences were searched against *M. gryphiswaldense* MSR-1 Uniprot database using PeaksOnline engine. The raw peak area for each protein was quantified by the software PeaksOnline X build 1.8, and then the relative quantitative analysis of the target protein was carried out.

Intracellular ROS level

ROS level was determined using a Reactive Oxygen Species Assay Kit (Solarbio; China) based on 2′,7′-dichlorofluorescin diacetate (DCFH-DA). Harvested MSR-1 cells were incubated with 10 μM cell-permeable DCFH-DA for 30 min at 37°C in the dark, washed 3x with PBS (pH 7.4), and analyzed by quantitative spectrofluorometry

(excitation wavelength 488 nm, emission wavelength 525 nm).

Supplementary Figure S1

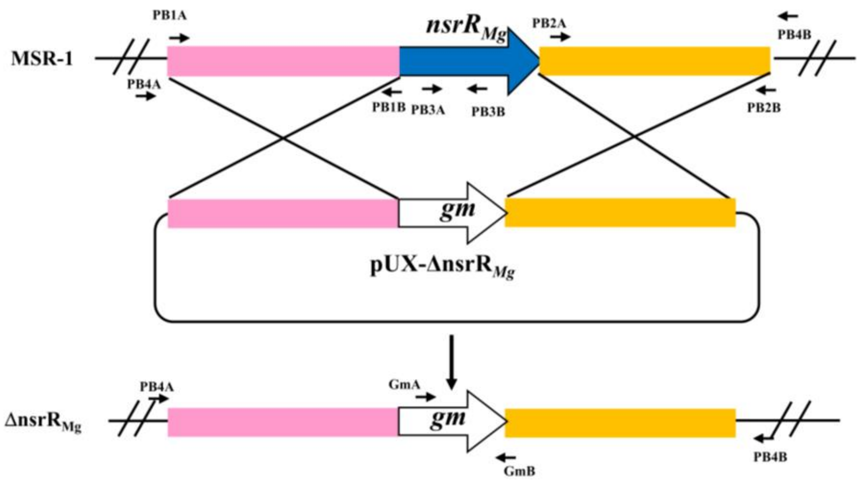

Figure S1. Strategy for $nsrR_{Mg}$ deletion in MSR-1 (schematic). Large arrows: genes and their directions. Small arrows: positions of primers. Blocks: homologous exchange regions used for gene deletion.

Supplementary Figure S2

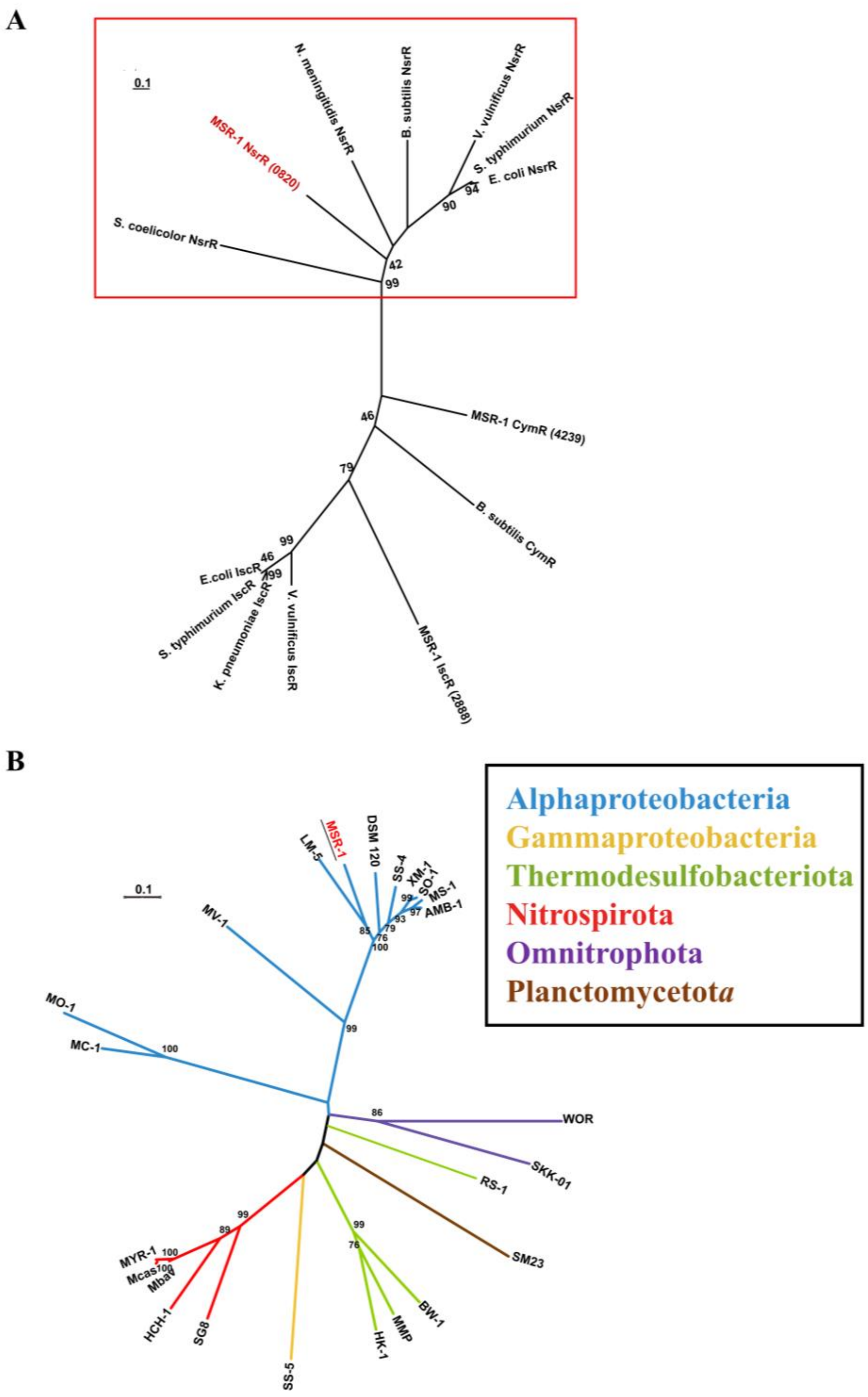

Figure S2. Phylogenetic analysis of NsrR. (**A**) Phylogenetic tree of Rrf2 family transcriptional regulators. The tree was constructed based on NsrR amino acid sequences from *M. gryphiswaldense* MSR-1, *E. coli*, *B. subtilis*, *Streptomyces coelicolor*, *S. typhimurium*, *Vibrio vulnificus*, and *Neisseria meningitidis*, IscR

sequences from *M. gryphiswaldense* MSR-1, *E. coli*, *S. typhimurium*, *V. vulnificus*, and *Klebsiella pneumoniae*, and CymR sequences from *M. gryphiswaldense* MSR-1, *B. subtilis*. (**B**) Phylogenetic tree of MTB NsrR. MTB from various bacterial classes used for construction of the tree are as follows. From Alphaproteobacteria: MSR-1, *Magnetospirillum magneticum* AMB-1, *Magnetospirillum* sp. XM-1, *Magnetospirillum molischianum* DSM 120, *Magnetospirillum* sp. SS-4, *Magnetospirillum* sp. LM-5, *Magnetospirillum magnetotacticum* MS-1, *Magnetococcus marinus* MC-1, *Magnetovibrio blakemorei* MV-1, *Magnetospirillum* sp. SO-1, *Magnetospira* sp. QH-2. From Gammaproteobacteria: strain SS-5. From Thermodesulfobacteriota: *Candidatus* [*Ca.*] *Desulfamplus magnetomortis* BW-1, *Desulfovibrio magneticus* RS-1, *Ca. Magnetoglobus multicellularis* (MMP). From Nitrospirota: *Ca. Magnetobacterium bavaricum* (Mbav), *Ca. Magnetobacterium casensis* (Mcas), *Ca. Magnetominusculus xianensis* HCH-1, *Ca. Magnetobacterium casensis* MYR-1, *Nitrospira bacterium* SG8_35_4. From Omnitrophota: *Ca. Omnitrophus magneticus* SKK-01, *Omnitrophica WOR_2 bacterium* GWA2_45_18. From Planctomycetota: *Planctomycetes bacterium* SM23_25. Scale bars represent 10% sequence divergence, and bootstrap values are expressed as percentages.

Supplementary Figure S3

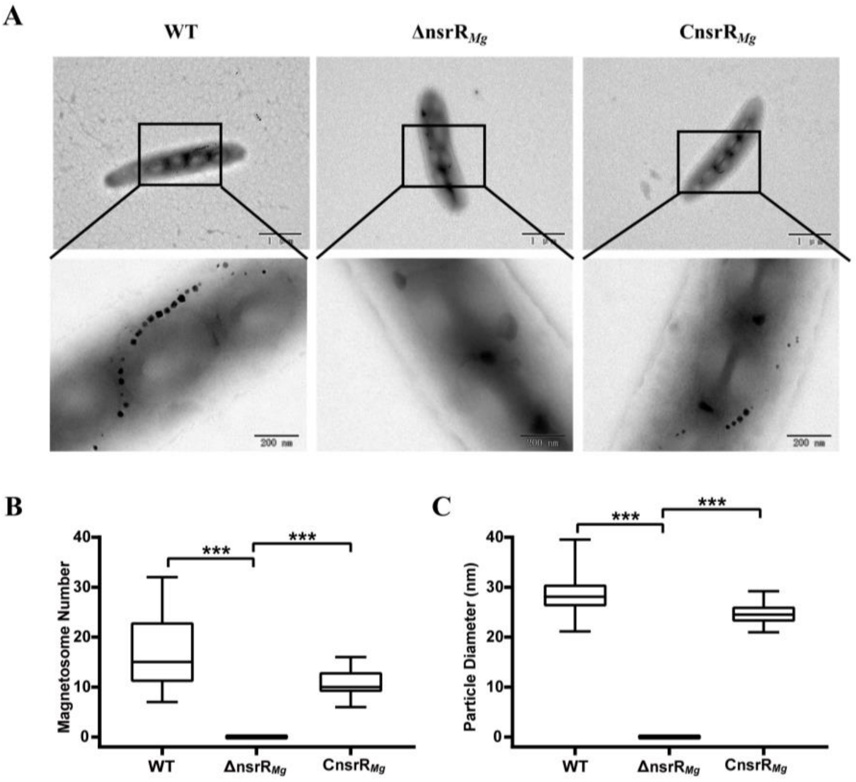

Figure S3. TEM analysis of MSR-1 strains WT, ΔnsrR$_{Mg}$ and CnsrR$_{Mg}$ cultured in nitrate medium (NH_4Cl in mSLM was replaced with $NaNO_3$) for 24 h. (**A**) TEM images with progressive magnification. Scale bars: 1 μm and 200 nm. (**B**) Box-plot charts of magnetosome numbers for WT, ΔnsrR$_{Mg}$, and CnsrR$_{Mg}$ (each n=35). (**C**) Magnetosome sizes for WT (n=593), ΔnsrR$_{Mg}$ (n=0), and CnsrR$_{Mg}$ (n=377). Error bars: mean ± SD from three biological replicates. ***$p< 0.001$, based on unpaired two-tailed Student's *t*-test.

Supplementary Figure S4

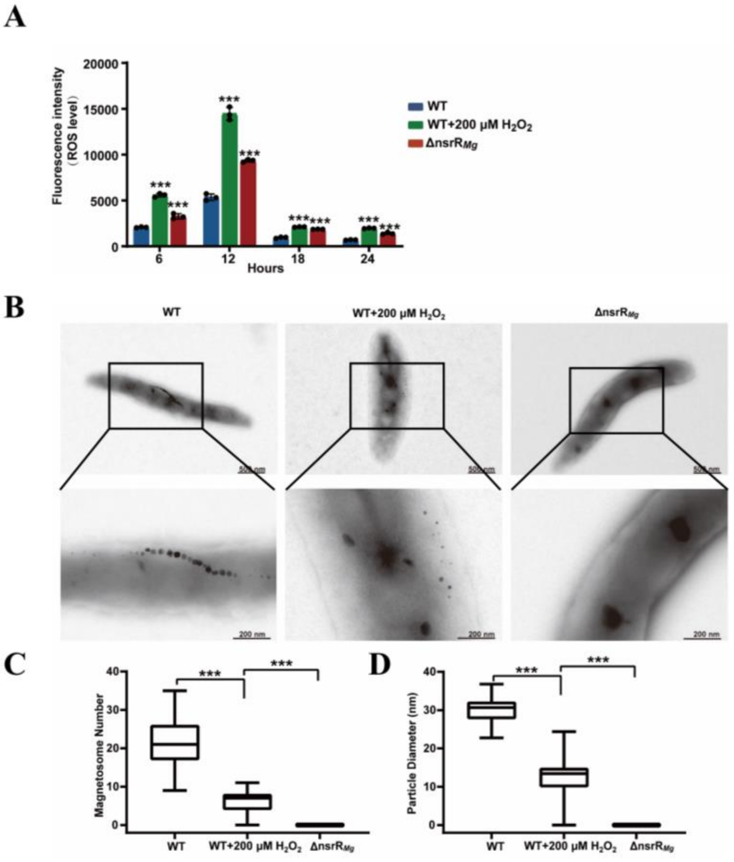

Figure S4. Effect of ROS on magnetosome formation. (**A**) Intracellular ROS levels in WT, WT treated with 200 µM H_2O_2, and ΔnsrR$_{Mg}$ cultured in mSLM. (**B**) TEM images of samples cultured for 24 h with progressive magnification. Scale bars: 500 and 200 nm. (**C**) Box-plot charts of magnetosome numbers for WT, WT treated with 200 µM H_2O_2, and ΔnsrR$_{Mg}$ (each n=35). (**D**) Magnetosome sizes for WT (n=735), WT treated with 200 µM H_2O_2 (n=305), and ΔnsrR$_{Mg}$ (n=0). Error bars: mean ± SD from three biological replicates. ***p< 0.001, based on unpaired two-tailed Student's *t*-test.

Supplementary Figure S5

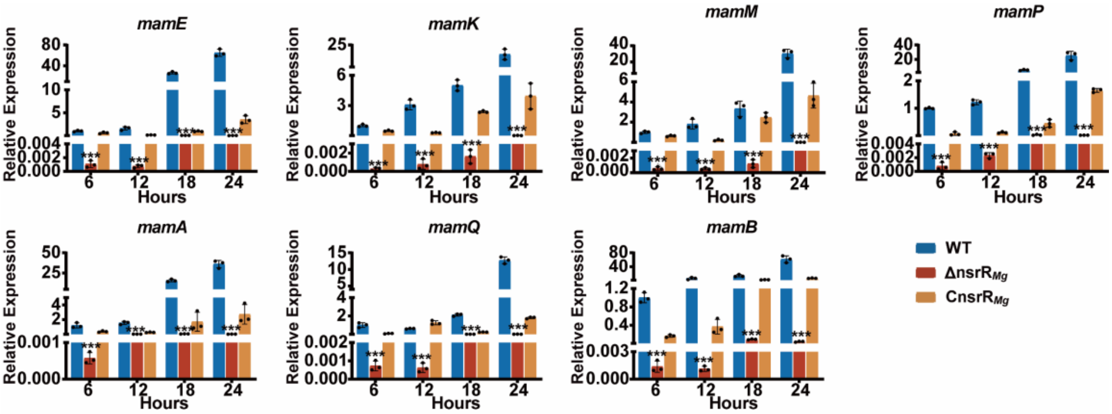

Figure S5. qRT-PCR analysis of seven conserved genes of *mamAB* operon in WT, ΔnsrR$_{Mg}$, and CnsrR$_{Mg}$ cultured in mSLM. Transcription level of each gene was expressed relative to that of WT at 6 h, defined as 1. Error bars: mean ± SD from three biological replicates. ***$p < 0.001$, based on unpaired two-tailed Student's *t*-test.

Supplementary Figure S6

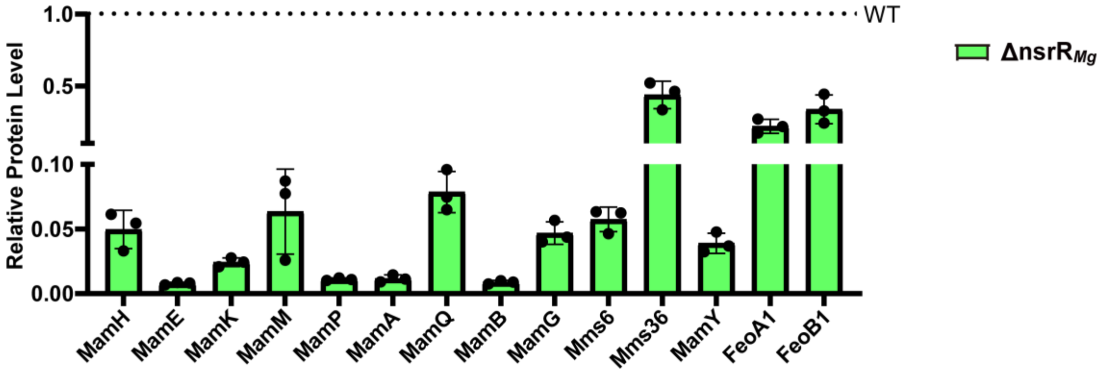

Figure S6. Relative levels of magnetosome-associated proteins in WT and ΔnsrR$_{Mg}$ quantified by mass spectrometry experiments. Protein samples were prepared from WT and ΔnsrR$_{Mg}$ cultured in mSLM for 12 h. For each protein, value in ΔnsrR$_{Mg}$ was calculated relative to WT level, defined as 1. Error bars: mean ± SD from three biological replicates.

Supplementary Figure S7

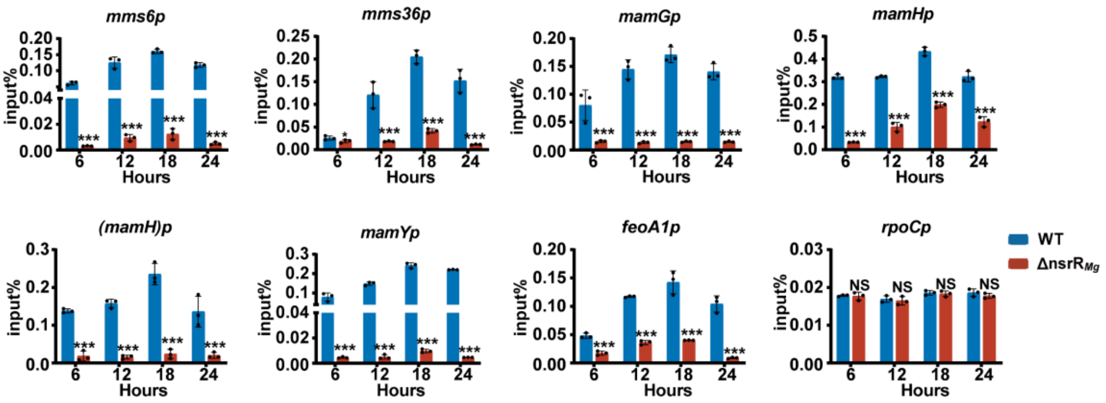

Figure S7. *In vivo* ChIP-qPCR assays of NsrR$_{Mg}$ binding to target promoters of MAI genes. Anti-NsrR$_{Mg}$ antibody was used to immunoprecipitate NsrR$_{Mg}$-DNA complexes from WT and ΔnsrR$_{Mg}$ cultured in mSLM for the indicated times. The *y*-axis indicates relative binding level of NsrR$_{Mg}$ on each site, determined by recovery of target DNA with anti-NsrR$_{Mg}$ antibody. Error bars: mean ± SD from three biological replicates. *$p < 0.05$, ***$p < 0.001$, NS: no significant difference, based on unpaired two-tailed Student's *t*-test.

Supplementary Figure S8

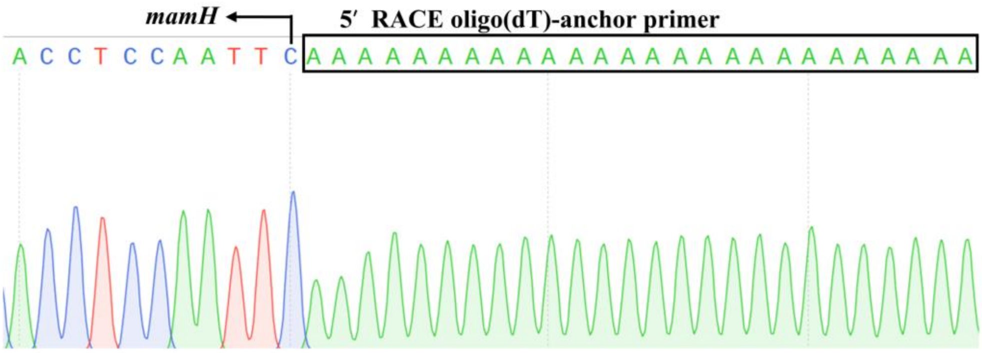

Figure S8. Determination of *mamH* TSS by 5' RACE. Box: complementary sequence of oligo (dT)-anchor primer. Bent arrow: complementary base of TSS.

Supplementary Figure S9

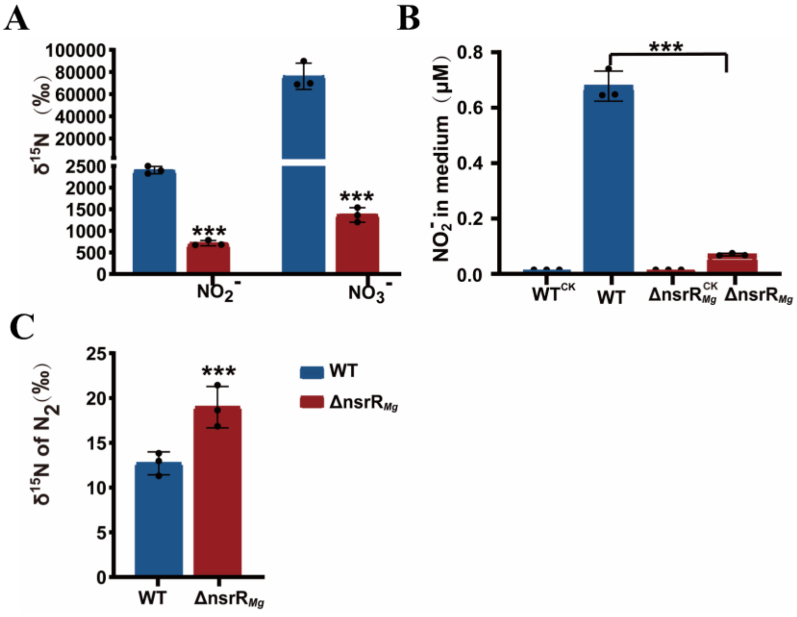

Figure S9. Nitrification-denitrification metabolic pathway in MSR-1. (**A**) Relative abundance of ^{15}N ($\delta^{15}N$) of NO^{2-} and of NO^{3-} produced by WT and ΔnsrR*Mg* during 18 h culture in mSLM with isotopically labeled $^{15}NH_4Cl$ (10% ^{15}N). (**B**) Nitrite content in medium following 18 h growth. Cells were grown in mSLM; control was medium inoculated with same amount of bacterial liquid at 0 h. (**C**) $\delta^{15}N$ of N_2 produced by WT and ΔnsrR*Mg* during 18 h culture in mSLM with isotopically labeled $^{15}NH_4Cl$ and $Na^{15}NO_3$. Error bars: mean ± SD from three biological replicates. ***$p < 0.001$, based on unpaired two-tailed Student's *t*-test.

Supplementary Figure S10

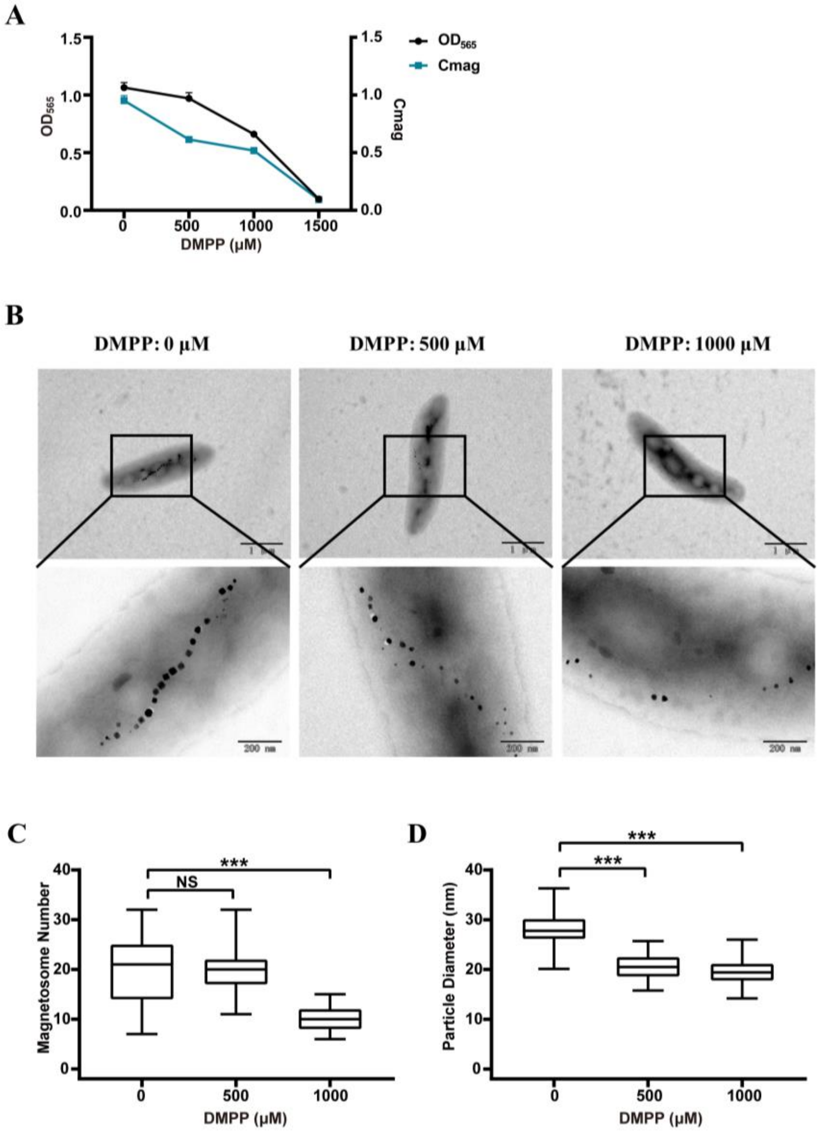

Figure S10. Effects of DMPP on cell growth and magnetosome formation. (**A**) Growth (OD_{565}) and Cmag of WT cultured for 24 h in mSLM with indicated DMPP concentrations. (**B**) TEM images with progressive magnification. Scale bars: 1 μm and 200 nm. (**C**) Box-plot charts of magnetosome numbers for WT treated with 0, 500, or 1000 μM DMPP (each n=35). (**D**) Magnetosome sizes for WT treated with 0 μM (n=696), 500 μM (n=684), or 1000 μM (n=377) DMPP. Error bars: mean ± SD from three biological replicates. ***$p < 0.001$, NS: no significant difference, based on unpaired two-tailed Student's *t*-test.

Supplementary Figure S11

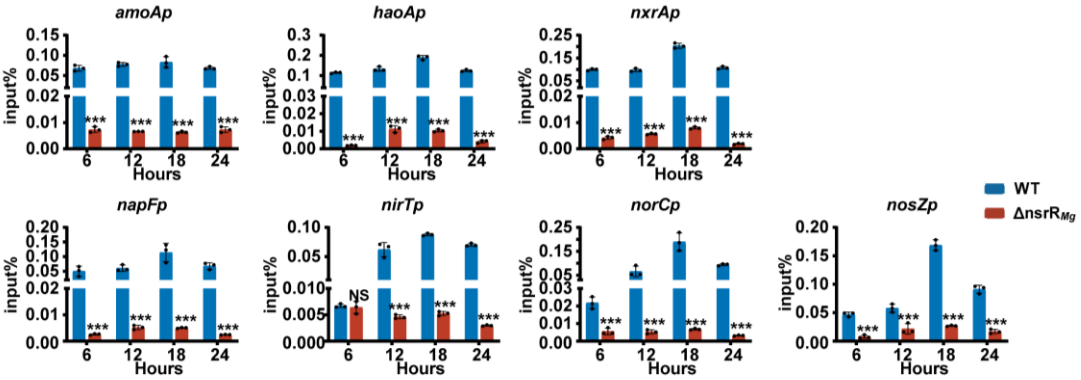

Figure S11 *In vivo* ChIP-qPCR assays of NsrR$_{Mg}$ binding to target promoters of nitrification and denitrification genes. Notations as in Supplementary Figure S7.

Supplementary Figure S12

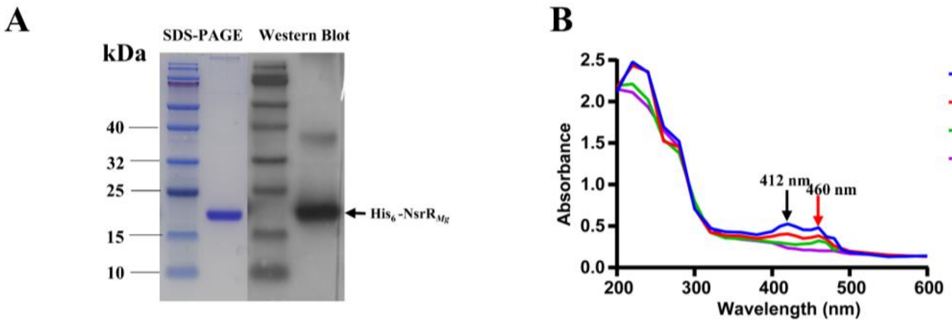

Figure S12. Effect of O_2 on degradation of the Fe-S cluster in His$_6$-NsrR$_{Mg}$. (**A**) SDS-PAGE and Western blot analysis of the anaerobically purified His$_6$-NsrR$_{Mg}$. (**B**) Absorption spectra of the anaerobically purified His$_6$-NsrR$_{Mg}$ after exposure to air for 0 to 4 h.

Supplementary Figure S13

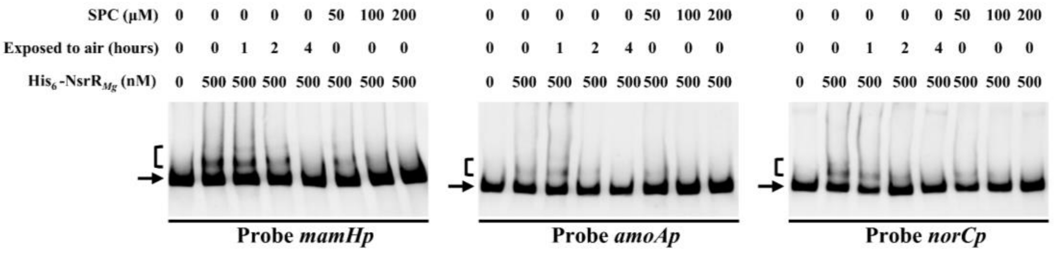

Figure S13. Effect of O_2 on NsrR$_{Mg}$ interaction with target promoters *mamHp*, *amoAp*, and *norCp*. EMSAs were performed with NsrR$_{Mg}$ (anaerobically purified), NsrR$_{Mg}$ exposed to air (1-4 h), and NsrR$_{Mg}$ with SPC at indicated concentrations.

Supplementary Figure S14

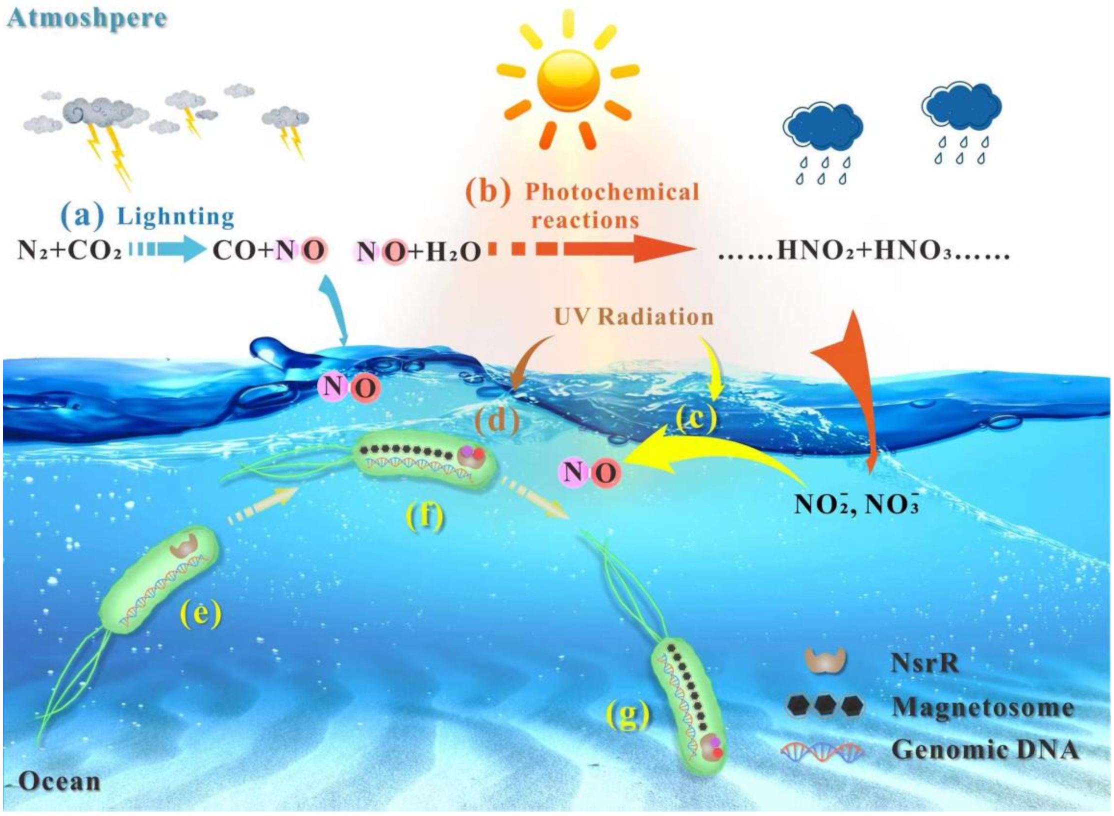

Figure S14. Proposed model of MTB behavior on early Earth prior to GOE. (**A**) During the Archean eon (3.8-2.5 Gyr ago), NO was generated by lightning strikes from CO_2 and N_2 in the atmosphere (sky blue arrows). (**B**) Photochemical reactions involving NO and water vapor generated various acids (*e.g.*, HNO_2, HNO_3), which were then transferred from the atmosphere to the ocean by rain (orange arrows). (**C**) NO_2^- at the ocean surface was converted to NO by UV radiation (yellow arrows). (**D**) Strong UV radiation at the ocean surface was potentially damaging to MTB and other Archean microorganisms (brown arrow). (**E**) However, it was necessary for MTB to move toward the ocean surface to perform nitrogen fixation for nutritional purposes. (**F**) Environmental NO was sensed by NsrR, which then activated magnetosome biosynthesis. (**G**) Formation of magnetosomes facilitated downward orientation and more efficient swimming away from nitrosative stress and UV radiation.

Supplementary Figure S15

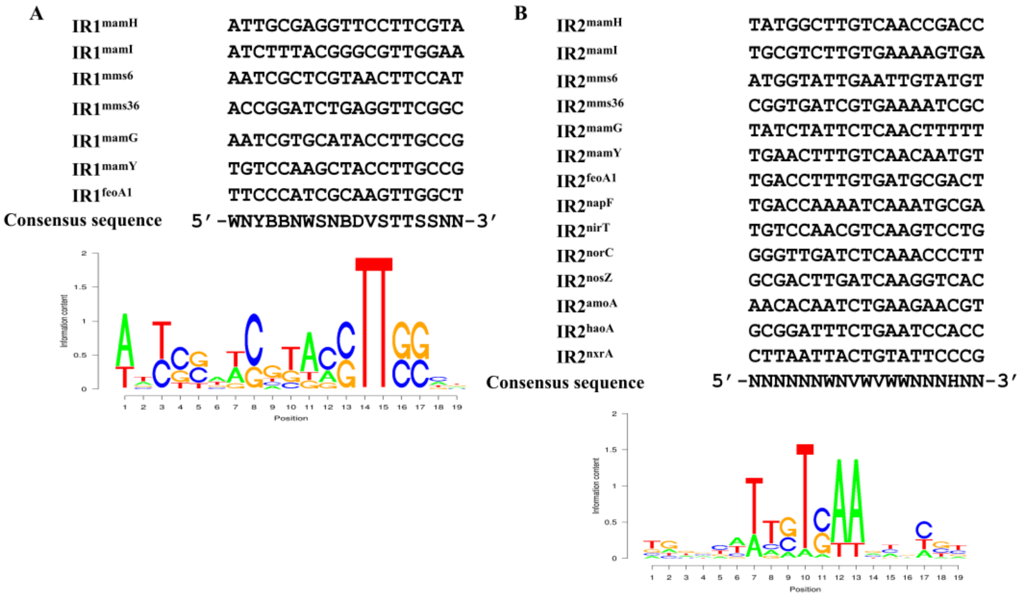

Figure S15. Analysis of consensus NsrR$_{Mg}$-binding sequences by WebLogo program. In the sequence logo of NsrR$_{Mg}$-binding consensus, appearance frequency of a particular base is proportional to height of corresponding letter.

Table S1. Strains and plasmids used in this study.

Strain or plasmid	Description	Source or reference
Magnetospirillum gryphiswaldense		
MSR-1	Wild-type strain, Nx^r	DSM 6361
ΔnsrR$_{Mg}$	*nsrR*$_{Mg}$ deletion mutant, Nx^r, Gm^r	This study
CnsrR$_{Mg}$	*nsrR*$_{Mg}$ complemented strain, Nx^r, Gm^r, Km^r	This study
ΔmamE	*mamE* deletion mutant, Nx^r	Yang et al. (2010)
Escherichia coli		
DH5α	General cloning host	Novagen
S17-1	The donor strain of conjugation	Novagen
S17-1-ΔnsrR$_{Mg}$	S17-1 containing pUX-19-*ΔnsrR*$_{Mg}$, Km^r, Gm^r	This study
BL21 (DE3)	Host for protein overexpression	Novagen
BL21-NsrR$_{Mg}$	BL21 containing pET28a (+)-*nsrR*$_{Mg}$, Km^r	This study
**Plasmids**		
pMD18-T	Cloning vector, Amp^r	TaKaRa
pET-28a (+)	Vector for protein overexpression in *E. coli*, Km^r	Novagen
pUC-Gm	pUC19 derivative containing *aacC1* (encodes Gm), Amp^r, Gm^r	Schweizer (1993)
pUX-19	Suicide vector for *M. gryphiswaldense* MSR-1, Km^r	Zhang et al. (2005)
pUX-ΔnsrR$_{Mg}$	*nsrR*$_{Mg}$ deletion vector based on pUX-19, Km^r	This study
pET-28a-NsrR$_{Mg}$	NsrR$_{Mg}$ overexpression vector based on pET-28a (+), Km^r	This study
pBBR1MCS-2	Broad-host range *lacZ* promoter probe vector, Amp^r, Km^r	Kovach et al. (1995)
pMCS-CnsrR$_{Mg}$	*nsrR*$_{Mg}$ complemented vector based on pBBR1MCS-2, Km^r	This study
pACYC184	Protein expression vector with *tet* promoter in reporter system, Tet^r, Cpl^r.	Tahlan et al. (2007)
pNsrR$_{Mg}$	NsrR$_{Mg}$ expression vector based on pACYC184	This study
pCS26-*Pac*	Reporter vector containing *lux* operon from pZS, Km^r	Tahlan *et al.* (2007)
pOmamHp-lux	pCS26-*Pac* containing *mamHp*-controlled *lux* reporter	This study
pOmamIp-lux	pCS26-*Pac* containing *mamIp*-controlled *lux* reporter	This study
pOnorCp-lux	pCS26-*Pac* containing *norCp*-controlled *lux* reporter	This study

Table S2. Primers used in this study.

Primer	Sequence (5′-3′)	Description
PB1A	GAATTCAGCCGCCGAAATAGAAGGT	Amplification upstream of *nsrR*$_{Mg}$
PB1B	GGATCCCGTAATCCGTGTGAAGCGT	Amplification upstream of *nsrR*$_{Mg}$
PB2A	GGATCCTGATCGATGCCGAACGGGCA	Amplification downstream of *nsrR*$_{Mg}$
PB2B	GAGCTCTTGAAACGGGACAGGGTT	Amplification downstream of *nsrR*$_{Mg}$
PB3A	TGACAATGCCGATATGATGAGC	Confirmation of *nsrR*$_{Mg}$ mutant
PB3B	CCGAGATGAGCGAAAGCAAG	Confirmation of *nsrR*$_{Mg}$ mutant
PB4A	GCCATGGTTTCGATGCCTTC	Confirmation of *nsrR*$_{Mg}$ mutant
PB4B	GTTTTACCGGGTGTTGGTGC	Confirmation of *nsrR*$_{Mg}$ mutant
PB7A	AAGCTTTTATTGAAACCCTGCCTTCA	Expression of NsrR$_{Mg}$ protein
PB7B	GGATCCATGCGCCCGCTGCCCACCTT	Expression of NsrR$_{Mg}$ protein
PB5A	CATGCATTTCATGCGACTTGACCAGGG	Complementation of *nsrR*$_{Mg}$ in ΔnsrR$_{Mg}$
PB5B	GGATCCGGCGTTGGTCGTATTATCCTT	Complementation of *nsrR*$_{Mg}$ in ΔnsrR$_{Mg}$
PB6A	CGGGATCCATGCGACTGACGCTTCAC	Complementation of *nsrR*$_{Mg}$ in ΔnsrR$_{Mg}$
PB6B	TCTAGAGCAGGCTGACGACACTACAT	Complementation of *nsrR*$_{Mg}$ in ΔnsrR$_{Mg}$
GmA	AGAACCTTGACCGAACGCA	Validation of Gm gene inserted in mutant
GmB	CGATCTCGGCTTGAACGAATT	Validation of Gm gene inserted in mutant
QrpoC-F	ATCTGGTCTACCGCCATTG	qRT-PCR for *rpoC* gene
QrpoC-R	CCTTGCCGAACGAAATACC	qRT-PCR for *rpoC* gene
QmamH-F	GCAGTCAATGCCAATGTGC	qRT-PCR for *mamH* gene
QmamH-R	CCAACCCAGGGACTTAGCG	qRT-PCR for *mamH* gene
QmamI-F	ATGCCAAGCGTGATTTTCGG	qRT-PCR for *mamI* gene
QmamI-R	GTTAGGGTCTGAGTTCGCCC	qRT-PCR for *mamI* gene
QmamE-F	CGCAATAGCGTGGTTAGCG	qRT-PCR for *mamE* gene
QmamE-R	TGAAGCCGTCATTGCGG	qRT-PCR for *mamE* gene
QmamM-F	ATGCCGTCCAGATCCTGTTG	qRT-PCR for *mamM* gene
QmamM-R	TAATGGGCGCCGATGATACC	qRT-PCR for *mamM* gene
QmamA-F	GCCTATCCGTGGCGAAGAA	qRT-PCR for *mamA* gene
QmamA-R	TCGGCATCGTAAACCTGCT	qRT-PCR for *mamA* gene
QmamB-F	AGGTCGTGTGGTGGGCAT	qRT-PCR for *mamB* gene
QmamB-R	CGCTCATCCGCAGGCTTA	qRT-PCR for *mamB* gene
QmamP-F	TTGTCATTGGGCGGCAG	qRT-PCR for *mamP* gene
QmamP-R	ATTGGGCAAGGGCGACT	qRT-PCR for *mamP* gene
QmamQ-F	GCCAGTTCGGTCGATAAGGT	qRT-PCR for *mamQ* gene
QmamQ-R	CCTCGAAATTGGAGCGCTTG	qRT-PCR for *mamQ* gene
QmamK-F	GAATACGAATGTGGCCTGC	qRT-PCR for *mamK* gene
QmamK-R	AAAATCTCGATGCTTTCAATGAT	qRT-PCR for *mamK* gene
QmamO-F	CGAGGACGACTACTTCCGTG	qRT-PCR for *mamO* gene
QmamO-R	GACGGAATGCAGGGTCGTAA	qRT-PCR for *mamO* gene
QmamG-F	ATGATCAAGGGCATCGCGG	qRT-PCR for *mamG* gene
QmamG-R	GAGAAGAACGGGACCCCAAG	qRT-PCR for *mamG* gene
Qmms6-F	GGTTGGCGTTGGGAAGGT	qRT-PCR for *mms6* gene

Qmms6-R	CATCGCTCTGTGCCGCTT	qRT-PCR for *mms6* gene
Qmms36-F	GCTGTCCTGGATAGGTGACG	qRT-PCR for *mms36* gene
Qmms36-R	AAACACCAGCCGCCTCTTTA	qRT-PCR for *mms36* gene
QmamY-F	GAGGAGCCCGCATCGTAT	qRT-PCR for *mamY* gene
QmamY-R	AGAGCAATCGGAAGTGAAATGG	qRT-PCR for *mamY* gene
QfeoA1-F	CCTTGCCGACCTTTGTCC	qRT-PCR for *feoA1* gene
QfeoA1-R	TCAGGCTGTAGATGCGGG	qRT-PCR for *feoA1* gene
QfeoB1-F	GAGGGCATGTTCCACGATCT	qRT-PCR for *feoB1* gene
QfeoB1-R	CATGGGGATCAGGGTAGTGC	qRT-PCR for *feoB1* gene
QnapF-F	TGATGTCGCACAGCCTTAG	qRT-PCR for *napF* gene
QnapF-R	TGATGTCGCACAGCCTTAG	qRT-PCR for *napF* gene
QnirT-F	CCATTCACTACACCAACCGTTC	qRT-PCR for *nirT* gene
QnirT-R	ATGGCAGTTGCGGCATTC	qRT-PCR for *nirT* gene
QnorC-F	CGGTGTTCGTTGCCTTGA	qRT-PCR for *norC* gene
QnorC-R	CAGACATTGCCCAGTTCCG	qRT-PCR for *norC* gene
QnosZ-F	TCGCCACGGTGTCCTTT	qRT-PCR for *nosZ* gene
QnosZ-R	ATCACCTGACCGCTTTGGC	qRT-PCR for *nosZ* gene
QamoA-F	TGTTCGCCGGTATGATCCTG	qRT-PCR for *amoA* gene
QamoA-R	CGATCCCATGGATTGGCTGA	qRT-PCR for *amoA* gene
QhaoA-F	AACCAAGCTGAACGAACCA	qRT-PCR for *haoA* gene
QhaoA-R	TGATCGGTGACGCCATTGAA	qRT-PCR for *haoA* gene
EmamH-F	CAGTGACGAAGGCAAGCA	EMSA for *mamAB* operon
EmamH-R	AATTCACCTCCAATTCGCAC	EMSA for *mamAB* operon
EmamI-F	GTTCTGTTCTCGGCGCATTT	EMSA for *mamAB* operon
EmamI-R	CCAGCAATCCAATGGCAAGC	EMSA for *mamAB* operon
EmamGFDC-F	TGGAGTCCAGTCAGCCCTACC	EMSA for *mamGFDC* operon
EmamGFDC-R	GCGATGCCCTTGATCATCTG	EMSA for *mamGFDC* operon
EmamXY-F	ACCAAGAACAGGAACACCC	EMSA for *mamXY* operon
EmamXY-R	CGACATATACGATGCGGGCT	EMSA for *mamXY* operon
Emms6-F	CGTGCTTACTCTTTGGGATT	EMSA for *mms6* operon
Emms6-R	AAACATCAGCCTCACCAGA	EMSA for *mms6* operon
Emms36-F	TCCATGGGGGTCCTGGTATT	EMSA for *mms6* operon
Emms36-R	CGGTGCCTTTTCGTTGATGT	EMSA for *mms6* operon
ErpoC-F	TGAAGGAAGCCAAGGACCT	EMSA for *rpoC* promoter
ErpoC-R	CGAGGGACGG GTCAAATCCC	EMSA for *rpoC* promoter
EnapF-F	CGGCGGTCAAGAAGATGA	EMSA for *nap* operon
EnapF-R	GAGTGCGCCCGAACAAGG	EMSA for *nap* operon
EnirT-F	AAGCAGCAGGGCGTTCCT	EMSA for *nir* operon
EnirT-R	AGTATTTTCATTTTGGACA	EMSA for *nir* operon
EnorC-F	AACCGATCTCATCGGCGAA	EMSA for *nor* operon
EnorC-R	TACCGCCATAAAAGATATT	EMSA for *nor* operon
EnosZ-F	AGACGTCGGGGCAGAAGGT	EMSA for *nos* operon
EnosZ-R	AGCGCGCCAAAGGACACCGT	EMSA for *nos* operon
EmamH-I-F	CAGTGACGAAGGCAAGCA	EMSA for *mamHp*-I

EmamH-I-R	CGAAGGAACCTCGCAATGT	EMSA for *mamHp*-I
EmamH-II-F	ACATTGCGAGGTTCCTTCG	EMSA for *mamHp*-II
EmamH-II-R	AATTCACCTCCAATTCGCAC	EMSA for *mamHp*-II
EmamH-III-F	GATGGCGCAAAGATGTGACG	EMSA for *mamHp*-III
EmamH-III-R	CGAAGGAACCTCGCAATGTG	EMSA for *mamHp*-III
EmamH-IV-F	ACATTGCGAGGTTCCTTCGT	EMSA for *mamHp*-IV
EmamH-IV-R	AATTCACCTCCAATTCGCAC	EMSA for *mamHp*-IV
mamH-sp1	TGACCCGCGAAACGTAGTAG	Identification of *mamH* TSS
mamH-sp2	ATCAAGGACCTCGGTCACCA	Identification of *mamH* TSS
mamH-sp3	CGGCTGAATAGCAACGACGA	Identification of *mamH* TSS
PB27A	GATATCTCCATTTACGCCCTTCAGCA	Amplification of NsrR$_{Mg}$
PB27 B	GGATCCACAACGGAATACCGCTTAGG	Amplification of NsrR$_{Mg}$
PB31A	CTCGAGCGTCTGGGTCATAGGGTCAG	Amplification of promoter in pOnorCp-lux
PB31B	GGATCCGCTTTCGTCAGTGTCTCGGA	Amplification of promoter in pOnorCp-lux
PB21A	GATATCCAGGTGAGTGATGCGGTTGA	Amplification of promoter in pOmamHp-lux
PB21B	GGATCCGCTTTATGGCGAACGCTATC	Amplification of promoter in pOmamHp-lux
PB22A	GTACTGCCGGGCCTCTTG	Confirmation of pNsrR$_{Mg}$
PB22B	GGTGATGTCGGCGATATAGG	Confirmation of pNsrR$_{Mg}$
PB23A	ATAGGCGTATCACGATTCCC	Confirmation of pOnorCp-lux
PB23B	TGCCATCCATTTTGCGGCC	Confirmation of pOnorCp-lux
PB24A	CCGGGCCTCTTGCGGGATATCCGGTTG AACGGGTCAGGC	Amplification of promoter in pOmamIp-lux
PB24B	TGCGTCCGGCGTAGAGGATCCTTATGG CTGCCGGTGGACT	Amplification of promoter in pOmamIp-lux

Table S3. NsrR$_{Mg}$ putative targets involved in nitrogen metabolism, iron metabolism, energy metabolism, or antioxidant function.

#	Accession number	Gene	Function	Score
**Nitrogen metabolism**				
1	MGMSRv2_2046	*dnrA*	TR	12,4
2	MGMSRv2_3659		nitronate monooxygenase	11.8
3	MGMSRv2_1402	*nirT*	nitrite reductase	11.8
4	MGMSRv2_3858	*haoA*	hydroxylamine oxidoreductase	11.6
5	MGMSRv2_1717	*norC*	NO reductase	10.7
6	MGMSRv2_3360	*amoA*	ammonia monooxygenase	10.4
7	MGMSRv2_2946	*fnr*	TR	10.2
8	MGMSRv2_3298	*amtB*	ammonium transporter	10.2
9	MGMSRv2_2007	*napF*	nitrate reductase	10.1
10	MGMSRv2_1430	*nosZ*	nitrous-oxide reductase	9.7
11	MGMSRv2_0154	*nxrA*	nitrite oxidoreductase	8.8
12	MGMSRv2_0185	*nrtA*	nitrate transporter, periplasmic nitrate binding protein	8.2
13	MGMSRv2_0181	*nasD*	NO reductase	8.2
**Iron metabolism**				
14	MGMSRv2_2313	*feoA1*	Fe^{2+} transport protein A	13.3
15	MGMSRv2_1033	*fpr*	ferredoxin--NADP reductase	13.1
16	MGMSRv2_3124	*fdx2*	ferredoxin--NADP reductase	12.5
17	MGMSRv2_3137	*fur*	iron-response regulator	10.6
18	MGMSRv2_1459	*apbC*	Fe-S cluster carrier protein	9.5
19	MGMSRv2_1443	*fdx1*	ferredoxin	9.4
20	MGMSRv2_3149	*irrB*	iron-response regulator	9.4
21	MGMSRv2_0314	*feoA2*	Fe^{2+} transport protein A	9.5
**Energy metabolism**				
22	MGMSRv2_0726	*iroA*	indolepyruvate oxidoreductase	14.4
23	MGMSRv2_1018	*pta*	phosphate acetyltransferase	13.2
24	MGMSRv2_1812	*ldh*	lactate dehydrogenase	12.8
**Antioxidant function**				
25	MGMSRv2_2107	*oxyR-like*	TR/redox-sensor	11.3
26	MGMSRv2_0756	*ahpC1*	peroxiredoxin	11.0
27	MGMSRv2_0756	*katE*	catalase/peroxidase	10.9
28	MGMSRv2_1221	*sodB*	superoxide dismutase	10.6
29	MGMSRv2_1805	*katG*	catalase/peroxidase	10.6

30	MGMSRv2_4250	*oxyR*	TR/redox-sensor	10.5

REFERENCES FOR SUPPLEMENTARY INFORMATION

1. Brenninkmeijer, C.A.M. and Röckmann, T. (1999) Mass spectrometry of the intramolecular nitrogen isotope distribution of environmental nitrous oxide using fragment-ion analysis. *Rapid Commun. Mass Spectrom.*, **13**, 2028-2033.
2. Edgar, R.C. (2004) MUSCLE: multiple sequence alignment with high accuracy and high throughput. *Nucleic Acids Res.*, **32**, 1792-1797.
3. Kumar, S., Stecher, G., Li, M., Knyaz, C., Tamura, K. and Battistuzzi, F.U. (2018) MEGA X: Molecular evolutionary genetics analysis across computing platforms. *Mol. Biol. Evol.*, **35**, 1547-1549.
4. Castresana, J. (2000) Selection of conserved blocks from multiple alignments for their use in phylogenetic analysis. *Mol. Biol. Evol.*, **17**, 540-552.
5. Guindon, S. and Gascuel, O. (2003) A simple, fast, and accurate algorithm to estimate large phylogenies by maximum likelihood. *Syst. Biol.*, **52**, 696-704.
6. Yan, H., Lu, X., Sun, D., Zhuang, S., Chen, Q., Chen, Z., Li, J. and Wen, Y. (2020) BldD, a master developmental repressor, activates antibiotic production in two *Streptomyces* species. *Mol. Microbiol.*, **113**, 123-142.
7. Yeku, O. and Frohman, M.A. (2011) Rapid amplification of cDNA ends (RACE). *Methods Mol. Biol.*, **703**, 107-122.
8. Grainger, D.C., Overton, T.W., Reppas, N., Wade, J.T., Tamai, E., Hobman, J.L., Constantinidou, C., Struhl, K., Church, G. and Busby, S.J. (2004) Genomic studies with *Escherichia coli* MelR protein: applications of chromatin immunoprecipitation and microarrays. *J. Bacteriol.*, **186**, 6938-6943.
9. Wiśniewski, J.R., Zougman, A., Nagaraj, N. and Mann, M. (2009) Universal sample preparation method for proteome analysis. *Nat. Meth.*, **6**, 359-362.
